# Structure of WzxE the lipid III flippase for Enterobacterial Common Antigen polysaccharide

**DOI:** 10.1098/rsob.240310

**Published:** 2025-01-08

**Authors:** Audrey Le Bas, Bradley R. Clarke, Tanisha Teelucksingh, Micah Lee, Kamel El Omari, Andrew M. Giltrap, Stephen A. McMahon, Hui Liu, John H. Beale, Vitaliy Mykhaylyk, Ramona Duman, Neil G. Paterson, Philip N. Ward, Peter J. Harrison, Miriam Weckener, Els Pardon, Jan Steyaert, Huanting Liu, Andrew Quigley, Benjamin G. Davis, Armin Wagner, Chris Whitfield, James H. Naismith

**Affiliations:** ^1^ Rosalind Franklin Institute, Harwell Campus, Didcot, UK; ^2^ Division of Structural Biology, University of Oxford, Roosevelt Drive, Oxford, UK; ^3^ Department of Molecular and Cellular Biology, University of Guelph, Guelph, Ontario, Canada; ^4^ Diamond Light Source, Harwell Science and Innovation Campus, Didcot, UK; ^5^ Department of Pharmacology, University of Oxford, Oxford, UK; ^6^ Department of Chemistry, Chemistry Research Laboratory, University of Oxford, Oxford, UK; ^7^ Biomedical Sciences Research Complex, North Haugh, University of St Andrews, St Andrews, UK; ^8^ Membrane Protein Laboratory, Diamond Light Source, Research Complex at Harwell, Didcot, UK; ^9^ Structural Biology Brussels, Vrije Universiteit Brussel (VUB), Pleinlaan 2, Brussels B-1050, Belgium; ^10^ VIB-VUB Center for Structural Biology, VIB, Pleinlaan 2, Brussels B-1050, Belgium

**Keywords:** flippase, oligosaccharides, lipid III, enterobacterial common antigen, membrane protein, cell wall

## Abstract

The enterobacterial common antigen (ECA) is conserved in Gram-negative bacteria of the *Enterobacterales* order although its function is debated. ECA biogenesis depends on the Wzx/Wzy-dependent strategy whereby the newly synthesized lipid-linked repeat units, lipid III, are transferred across the inner membrane by the lipid III flippase WzxE. WzxE is part of the Wzx family and required in many glycan assembly systems, but an understanding of its molecular mechanism is hindered due to a lack of structural evidence. Here, we present the first X-ray structures of WzxE from *Escherichia coli* in complex with nanobodies. Both inward- and outward-facing conformations highlight two pairs of arginine residues that move in a reciprocal fashion, enabling flipping. One of the arginine pairs coordinated to a glutamate residue is essential for activity along with the C-terminal arginine rich tail located close to the entrance of the lumen. This work helps understand the translocation mechanism of the Wzx flippase family.

**Figure:**
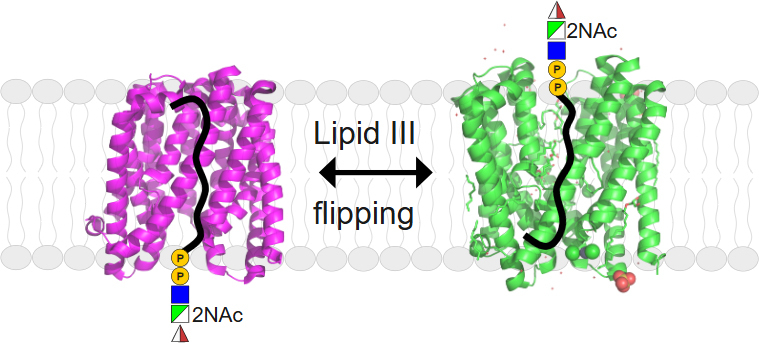

## 1. Introduction

**Figure 1.**
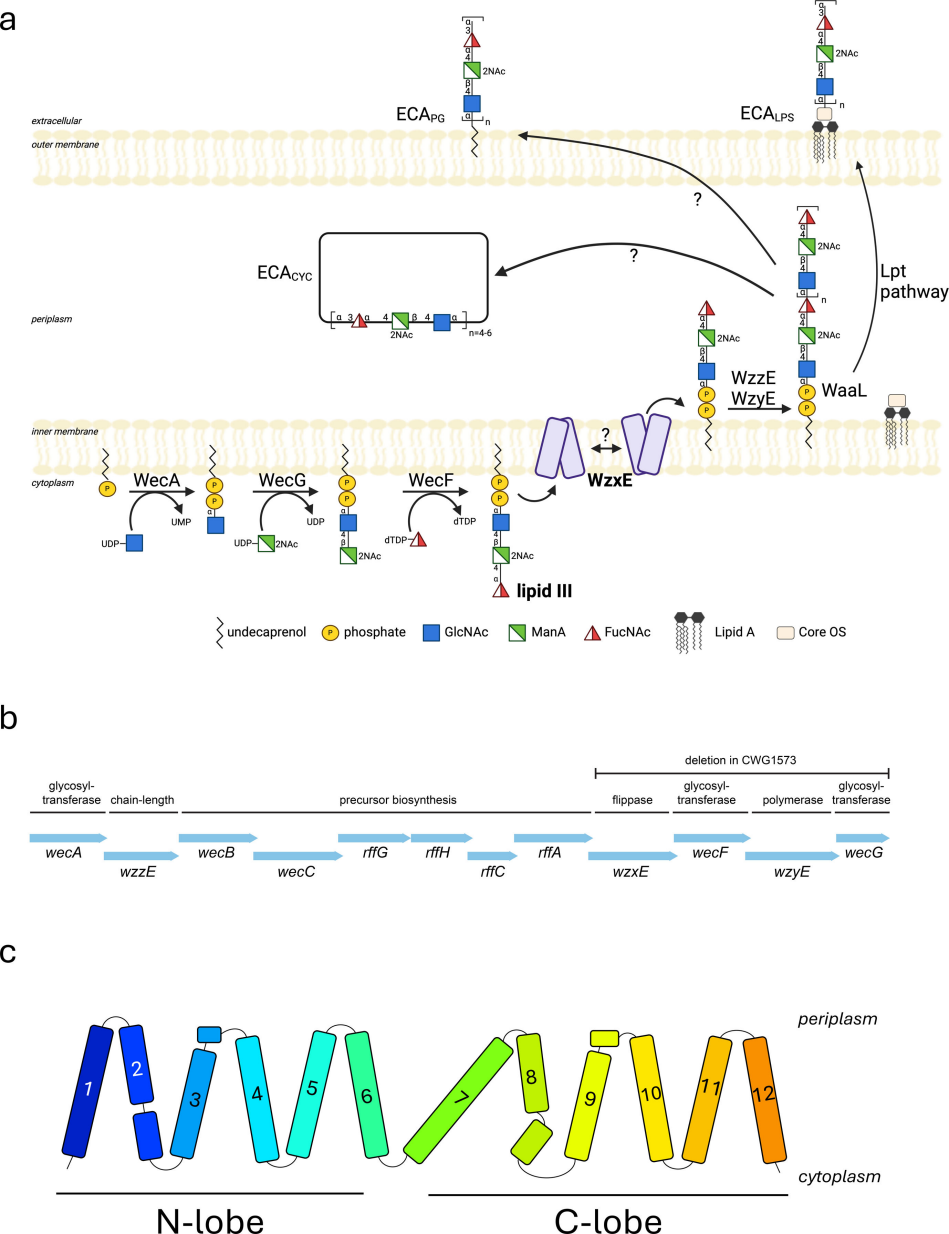
The lipid III flippase WzxE and the ECA pathway. (*a*) Schematic of the enterobacterial common antigen (ECA) pathway in *E. coli*. The lipid III synthesis is initiated by WecA which transfers a GlcNAc-P to und-P, followed by glycosyltransferases WecF and WecG that build the remainder of lipid III. WzxE flips the lipid III across the inner membrane and the O-antigen is polymerized by the complex WzyE–WzzE. The O-antigen is ligated by WaaL and exported to the outer membrane via the Lpt complex to form ECA_LPS_. The pathway for the ECA_PG_ and ECA_CYC_ is still under debate. This figure was created with BioRender.com (RRID:SCR_018361). (*b*) Gene cluster for the ECA pathway in *E. coli*. The *E. coli* CWG1573 strain, used here for the *in vivo* assay, contains a deletion of the genes *wzxE*, *wecF*, *wzyE*, *wecG*. (*c*) Schematic diagram of the topology of WzxE *E. coli*, with its 12 transmembrane α helices split into a N- and a C-lobe based on the WzxE original structure (PDB-ID: 9G97).

The outer surfaces of Gram-negative bacteria are coated with diverse glycoconjugates which are essential for survival and pathogenicity; carbohydrate polymers include lipopolysaccharides (LPS), capsular polysaccharides and exopolysaccharides [1,2]. Some of the structures are strain-specific and allow serological classifications, while others are conserved across species. Bacteria in the order *Enterobacterales* possess the glycoconjugate known as enterobacterial common antigen (ECA). ECA is composed of the repeating trisaccharide [-3-α-D-Fuc4NAc-(1,4)-β-D-ManNAcA-(1,4)-α-D-GlcNAc-1-] and exists in three different forms: ECA_LPS_ (corresponding to O14 in *E. coli* serology) and ECA_PG_ are expected to be surface exposed, but ECA_CYC_ is an unusual cyclic form found in the periplasm [3,4] (figure 1*a*). Although the different forms have been recognized for a long time and functions have been proposed, their precise physiological roles have not been definitively established.

ECA synthesis is mediated by a Wzy-dependent pathway using *‘en-bloc’* polymerization of lipid-linked repeat units (figure 1*a*). The membrane protein WecA [5] is a polytopic phosphoglycosyl transferase [6] that initiates synthesis by catalysing the transfer of a N-acetylglucosamine-1-phosphate (GlcNAc-1-P) onto the lipid carrier undecaprenol-phosphate (und-P) at the cytoplasmic face of the inner membrane [7,8]. The cytoplasmic glycosyltransferases, WecG and WecF, sequentially add an α-linked N-acetyl-D-mannosaminouronate (ManNAcA) and a 4-acetamido-4,6-dideoxy-D-galactose (Fuc4NAc) to complete the polyprenol-linked repeat unit [9–13]. The resulting lipid III (und-PP-GlcNAc-ManNAcA-Fuc4NAc) is then flipped across the membrane by the flippase WzxE [14,15]. The flipped trisaccharide is polymerized on the periplasmic side of the inner membrane by the integral membrane GT-C fold polymerase, WzyE [16], part of a transmembrane complex with the chain length regulator WzzE [17]. The terminal steps leading to the formation of periplasmic cyclic ECA_CYC_, usually composed of 4–6 repeat units [18], are uncertain. ECA_PG_ is proposed to be formed by the attachment of the ECA carbohydrate to a diacylglycerol, from phosphatidylglycerol, via a phosphodiester linkage [19] but how it gets to its proposed location in the outer membrane is still unknown. In some strains, a portion of the ECA is ligated to the lipid A-core oligosaccharide component of the LPS to create ECA_LPS_ and is exported to the outer membrane. Those two last steps are predicted to be mediated by the LPS O-antigen ligase (WaaL) [20] and the conserved lipopolysaccharide transport (Lpt) pathway [21].

The WzxE flippase is an essential part of this pathway and belongs to a larger family of Wzx proteins, which are representatives of multidrug and toxic compound extrusion (MATE) exporters. Wzx proteins translocate a range of lipid-linked O-antigen, capsular polysaccharide and exopolysaccharide repeat units across the bacterial inner membrane [22]. Many bacterial genomes encode different *wzx* genes associated with different glycan production systems which differ significantly from one another [23]. While their hydrophobicity, low sequence similarity and the lack of functional motifs have hindered progress on understanding these proteins, recent advances on the MurJ lipid II flippase [24,25] have begun to describe the Wzx mechanism. The initial proposal that Wzx is a flippase was suggested by showing accumulation of polyprenol-linked O-antigen intermediates in a *wzx*-deletion mutant [14]. Under conditions of overexpression, Wzx proteins can be somewhat promiscuous for the saccharide substrate, allowing functional exchange of homologs between species and systems and, in some cases, they can also translocate a truncated substrate: und-PP-GlcNAc [26,27]. WzxE can complement a *wzx*-deletion in the O-antigen biosynthesis pathway from *E. coli* O16 but only when the whole ECA cluster is deleted (figure 1*b*), hinting at a supramolecular complex which must be disrupted [28,29]. However, at chromosomal copy levels, these proteins appear to more stringently recognize the conserved und-PP-sugar of the substrate and show fidelity for the particular lipid-linked oligosaccharide [30]. The Wzx translocation mechanism remains unclear [23,31] but Wzx proteins are ATP-independent [15]. WzxE was identified as part of the ECA pathway in 2003 [15] and its specificity for lipid III has been well established [3].

Our understanding of WzxE (and Wzx proteins in general) is limited and no experimentally solved three-dimensional structures have been reported so far. Early studies proposed structural similarity to the NorM protein of the MATE family [32–36], a finding subsequently supported by an AlphaFold [37,38] model of WzxE (AF-P0AAA7-F1). The structure of the lipid II flippase MurJ, member of the MOP exporter family, was recently solved by X-ray crystallography [39–43] and strongly resembles the WzxE model. The MurJ and Wzx mechanisms of action are also thought to be similar, as recently shown by a functional exchange between MurJ and relaxed-specificity mutants of the flippase WzxC from the biosynthesis pathway for colanic acid exopolysaccharides [44]. The absence of Wzx structural information limits a deeper understanding of the export mechanism.

Here we present the first experimental structure of the lipid III flippase WzxE from *E. coli* crystallized in the lipidic cubic phase of monoolein in the presence a nanobody (Nb). The 2.4 Å resolution structure was determined prior to the release of the AlphaFold model. The structure reveals a monomer of 12 transmembrane helices (figure 1*c*), separated into two helical bundles, adopting an outward-facing conformation (open towards the periplasm) similar to the NorM protein from the MATE family [35]. Addition of a second nanobody enabled crystallization of the WzxE protein in an occluded inward-facing conformation, open towards the cytoplasm. An *in vivo* assay using a *wzxE*-deletion mutant of *E. coli* allowed the functional assessment of a series of WzxE mutants. Those results support a proposed mechanism for the translocation of lipid III across the membrane.

## 2. Material and methods

### 2.1. WzxE cloning and mutagenesis

Primers used for cloning and mutagenesis are described in table 1. The *wzxE* gene (NCBI gene ID 948294) from *E. coli* K-12 MG1655 was cloned into the pBAD-CTEVHIS_10_ vector, designed by Dr Huanting Liu. The DNA was amplified by PCR using Q5 High Fidelity DNA polymerase (NEB) from genomic DNA. The DNA fragment and the vector were cleaved using NcoI and XhoI (NEB) for 1 h at 37°C. The linearized vector and fragment were ligated with T4 DNA ligase (NEB) for 1 h at 20°C followed transformation of *E. coli* DH5⍺ (NEB). The *wzxE* mutants and non-tagged *wzxE* plasmids were obtained by site-directed mutagenesis, using the pBAD-wzxE-TEV-His_10_ plasmid as template and the Q5 polymerase (NEB) [45].

**Table 1.** Primers used in this study.

primer	sequence	description
WzxE-F	AAATTTCCATGGTGTCGTTGGCAAAAGCGTC	Fwd to amplify *wzxE* from *E. coli* K12 genome
WzxE-R	AAATTTCTCGAGTGCCCGCCTACGCCAGAGTAAAAAC	Rev to amplify *wzxE* from *E. coli* K12 genome
R44A-F	CAAATTTCGCCCAGTTGATTACCGTGCTCGGCGTGCTTGC	*wzxE* R44A Fwd mutagenesis primer
R44A-R	GTAATCAACTGGGCGAAATTTGCCGCCAGCCCAAGCCC	*wzxE* R44A Rev mutagenesis primer
V49A-F	GATTACCGCGCTCGGCGTGCTTGCCGGGGCTGG	*wzxE* V49A Fwd mutagenesis primer
V49A-R	GCCGAGCGCGGTAATCAACTGGCGGAAATTTGCCGC	*wzxE* V49A Rev mutagenesis primer
L53A-F	GGCGTGGCTGCCGGGGCTGGCATCTTTAACGGTGTAAC	*wzxE* L53A Fwd mutagenesis primer
L53A-R	CCCCGGCAGCCACGCCGAGCACGGTAATCAACTGGC	*wzxE* L53A Rev mutagenesis primer
Q74A-F	AATCCGGCACAGCTGCGCCGCGTGGTCGGCACTTC	*wzxE* Q74A Fwd mutagenesis primer
Q74A-R	GCACCAATCGCGACCAGTGTACTGGCCGCCGTCCAC	*wzxE* Q74A Rev mutagenesis primer
R77A-F	AACAGCTGGCCCGCGTGGTCGGCACTTCATCAGCGATG	*wzxE* R77A Fwd mutagenesis primer
R77A-R	CCACGCGGGCCAGCTGTTGCGGATTATCATGGTACTGG	*wzxE* R77A Rev mutagenesis primer
R78A-F	CTGCGCGCCGTGGTCGGCACTTCATCAGCGATGGTAC	*wzxE* R78A Fwd mutagenesis primer
R78A-R	CGACCACGGCGCGCAGCTGTTGCGGATTATCATGGTAC	*wzxE* R78A Rev mutagenesis primer
R77A-R78A-F	CTGGCCGCCGTGGTCGGCACTTCATCAGCGATGGTAC	*wzxE* R78A Fwd mutagenesis primer using *wzxE* R77A plasmid as template
R77A-R78A-R	CGACCACGGCGGCCAGCTGTTGCGGATTATCATGGTAC	*wzxE* R78A Rev mutagenesis primer using *wzxE* R77A plasmid as template
F90V-F	TACTTGGTGTCTCTACGCTGATGGCGCTGGTTTTTGTGC	*wzxE* F90V Fwd mutagenesis primer
F90V-R	GCGTAGAGACACCAAGTACCATCGCTGATGAAGTGCCG	*wzxE* F90V Rev mutagenesis primer
F98V-F	GCTGGTTGTTGTGCTGGCAGCTGCGCCAATCAGCC	*wzxE* F98V Fwd mutagenesis primer
F98V-R	CCAGCACAACAACCAGCGCCATCAGCGTAGAGAAACC	*wzxE* F98V Rev mutagenesis primer
R143A-F	AAGGCTTTGCCGATGCCGCAGGTAATGCGTTATCGCTG	*wzxE* R143A Fwd mutagenesis primer
R143A-R	CGGCATCGGCAAAGCCTTTCATCAGCGCCAGTAACAGG	*wzxE* R143A Rev mutagenesis primer
D210A-F	CAGCTGGGCTAACGGTCTGGCAGGGCAGTTGAGCG	*wzxE* R210A Fwd mutagenesis primer
D210A-R	GACCGTTAGCCCAGCTGGGTTTCAGATAGCTTAACGG	*wzxE* R210A Rev mutagenesis primer
R239A-F	TCATGATGGCTAAACTGCTGGCGGCGCAGTATAGCTGG	*wzxE* R239A Fwd mutagenesis primer and R44A–R239A Fwd mutagenesis primer using WzxE R44A template
R239A-R	CAGCAGTTTAGCCATCATGATGTAAGCAACAGGCAAGGTC	*wzxE* R239A Rev mutagenesis primer and R44A–R239A Rev mutagenesis primer using WzxE R44A template
D262A-F	ATTTCCGCTGCCTACCTGCAATTTATTACGGCATCGTTCAG	*wzxE* R262A Fwd mutagenesis primer
D262A-R	CAGGTAGGCAGCGGAAATACTGCTCACCCCTTGCCAG	*wzxE* R262A Rev mutagenesis primer
R282A-F	GTTGTCGGCGCTAACGGAAAAGCGCGATATCACCCG	*wzxE* R282A Fwd mutagenesis primer and R143A–R282A Fwd mutagenesis primer using WzxE R143A template
R282A-R	CCGTTAGCGCCGACAACGTGGGCAGCAAATATACGC	*wzxE* R282A Rev mutagenesis primer and R143A–R282A Rev mutagenesis primer using WzxE R143A template
D288A-F	CTAACGGAAAAGGCCGATATCACCCGGGAAGTGGTTAAATCG	*wzxE* D288A Fwd mutagenesis primer
D288A-R	GATATCGGCCTTTTCCGTTAGCCGCGACAACGTGGGCAG	*wzxE* D288A Rev mutagenesis primer
R413A-F	CTCTGGGCTAGGCGGGCACTCGAGGAAAACCTGTATTTTC	*wzxE* R413A Fwd mutagenesis primer
R413A-R	GCCCGCCTAGCCCAGAGTAAAAACACGCCACAACAAAGAG	*wzxE* R413A Rev mutagenesis primer
R414A-F	CTGGCGTGCGCGGGCACTCGAGGAAAACCTGTATTTTC	*wzxE* R414A Fwd mutagenesis primer
R414A-R	TGCCCGCGCACGCCAGAGTAAAAACACGCCACAACAAAG	*wzxE* R414A Rev mutagenesis primer
R415A-F	GCGTAGGGCGGCACTCGAGGAAAACCTGTATTTTCAGGG	*wzxE* R415A Fwd mutagenesis primer
R415A-R	CGAGTGCCGCCCTACGCCAGAGTAAAAACACGCCACAAC	*wzxE* R415A Rev mutagenesis primer
R413A-R414A-R415A-F	CTCTGGGCTGCGGCGGCACTCGAGGAAAACCTGTATTTTCAGG	*wzxE* R413A–R414A–R415A Fwd mutagenesis primer
R413A-R414A-R415A-R	GAGTGCCGCCGCAGCCCAGAGTAAAAACACGCCACAACAAAGAG	*wzxE* R413A–R414A–R415A Rev mutagenesis primer
WzxE-Stop-F	GCGGGCATAACTCGAGG AAAACCTGTATTTTCAGGGCG	*wzxE* stop codon insertion before His_10_ Fwd mutagenesis primer
WzxE-Stop-R	CCTCGAGTTATGCCCGCCTACGCCAGAGTAAAAACACG	*wzxE* stop codon insertion before His_10_ Rev mutagenesis primer
Nb10-strep-F	CAGGTGCTGGCAGTGGAGCGTCCGCGTGGAGTCATCCGCAATTCGAAAAATAGCACCACCATCACCATCACG	Nb10 Fwd mutagenesis primer to insert strep II tag and stop codon
Nb10-strep-R	CACTGCCAGCACCTGAACCCGCGCCTTTCTCGAACTGAGGATGGGACCAACTGGAGACGGTGACCTG	Nb10 Rev mutagenesis primer to insert strep II tag and stop codon
3262	CGTCAATCAGCGTACGGTAATTGCGACTTTGTTGAACTACTTTT CCTGATGTGTAGGCTGGAGCTGCTTC	Fwd to amplify Kanamycin^R^ cassette from pKD4 for λ red mutagenesis of *wzxE-wecG* region of SΦ874
3334	GCAGACAGGCGACGGAGTGACCACTCCGTCGCTTTACAAAGA GAGGAAAACATATGAATATCCTCCTTA	Rev to amplify Kanamycin^R^ cassette from pKD4 for λ red mutagenesis of *wzxE-wecG* region of SΦ874
3336	GGCTAGCAGGAGGAATTCACCATGGTGTCGTTGGCAAAAGCG	Fwd to amplify *wzxE* for Gibson assembly (paired with 3339)
3339	CAGTACGTGAATCAGTACAGTCATGCCCGCCTACGCCAGAG	Rev to amplify *wzxE* for Gibson assembly (paired with 3336)
3340	CTCTGGCGTAGGCGGGCATGACTGTACTGATTCACGTAC	Fwd to amplify *wecF wzyE wecG* for Gibson assembly (paired with 3341 or 3633)
3341	CTCATCCGCCAAAACAGCCAAGCTTTCATAGGTTGCCGGTGTAGTG	Rev to amplify *wecF wzyE wecG* for Gibson assembly (paired with 3340)
3559	CTGGCGTAGGGCGGCATGAATGG	*wzxE* R415A Fwd mutagenesis primer
3560	AGTAAAAACACGCCACAAC	*wzxE* R415A Rev mutagenesis primer
3561	ACTCTGGCGTGCGCGGGCATGAA	*wzxE* R414A Fwd mutagenesis primer
3562	AAAAACACGCCACAACAAAG	*wzxE* R414A Rev mutagenesis primer
3563	TTTACTCTGGGCTAGGCGGGCATGAATG	*wzxE* R413A Fwd mutagenesis primer
3564	AACACGCCACAACAAAGAG	*wzxE* R413A Rev mutagenesis primer
3633	TGACTGTACTGATTCACGTACTGGGATCG	Fwd to amplify *wecF wzyE wecG* for Gibson assembly with *wzxE* C-terminal Arg mutants (paired with 3341)
3632	CAGTACGTGAATCAGTACAGTCATGCGGCTGCCGCCCAGAGTAAAAACACGCCACAAC	Rev to amplify *wzxE* R414A-R415A triple mutant for Gibson assembly (paired with 3660)
3657	CAGTACGTGAATCAGTACAGTCATGCGGCTGCACGCCAGAGTAAAAACACGCCACAAC	Rev to amplify *wzxE* R414A/R415A mutant for Gibson assembly (paired with 3660)
3658	CAGTACGTGAATCAGTACAGTCATGCCCGTGCCGCCCAGAGTAAAAACACGCCACAAC	Rev to amplify *wzxE* R413A/R414A mutant for Gibson assembly (paired with 3660)
3659	CAGTACGTGAATCAGTACAGTCATGCGGCCCTCGCCCAGAGTAAAAACACGCCACAAC	Rev to amplify *wzxE* R413A/R415A mutant for Gibson assembly (paired with 3660)
3660	CCCGTTTTTTTGGGCTAGCAGGAGGAATTCACCATGTCGTTGGCAAAAGCGTCC	Fwd to amplify *wzxE* for Gibson assembly (paired with rev primers 3632 and 3657–3659)

The chromosomal region of the *E. coli* K-12 strain SΦ874 [46] encompassing *wzxE wecF wzyE* and *wecG* was replaced with a chloramphenicol-resistance cassette by lambda red mutagenesis [47], to produce CWG1573 (figure 1*b*). Deletion of the ECA genes and replacement with the resistance cassette was confirmed by whole genome Illumina sequencing (Seqcenter, Pittsburg, USA). The SΦ874 genome contains a large deletion encompassing the colanic acid and O-polysaccharide biosynthesis gene clusters, including the cognate *wzx* genes [46]. Plasmid pKD3 was used as a template for the chloramphenicol resistance cassette and pSIM6 was used to overexpress the lambda red recombination genes. Loss of ECA production was confirmed by the absence of ECA in immunoblots of the whole-cell lysates.

WzxE mutant derivatives were tested for biological activity by transforming CWG1573 with plasmids carrying *wecF*, *wzyE* and *wecG*. DNA fragments containing *wzxE* were amplified by PCR with KOD DNA polymerase (Novagen) from plasmids carrying the wild-type gene or from site-directed mutants. These were cloned by Gibson assembly (NEB) into linearized (EcoRI-HindIII) pBAD24 [48] together with a fragment containing *wecF*, *wzyE* and *wecG* amplified from SΦ874 genomic DNA. Unlike CWG1573, *E. coli* DH5α contains a functional chromosomal *wzxE* gene and this strain was used as the primary cloning host to mitigate any deleterious effects that could occur with cloning non-functional *wzxE* mutants. Fragments for Gibson Assembly of *wzxE* derivatives encoding the double and triple substitutions of R413, R414 or R415 were amplified directly from a plasmid containing wild-type *wzxE* using primers incorporating the mutated codons. Plasmid sequences were confirmed by DNA sequencing.

### 2.2. WzxE expression and purification

The recombinant pBAD-WzxE-TEV-His_10_ vector was transformed into *E. coli* C43(DE3) cells (Sigma-Aldrich) [49]. Transformants were grown in LB media at 37°C with 100 µg ml^−1^ ampicillin. WzxE protein production was induced by the addition of 0.2% arabinose at an OD_600nm_ of 1 and growth was continued for 3 h at 37°C. The cells were harvested at 5488 × g for 10 min, resuspended in 300 ml of Phosphate Buffered Saline, pH 7.4 (PBS) containing 25 µg ml^−1^ DNAse I and lysed using a cell disruptor (Cell Constant) at a pressure of 30 kPSI. The lysate was centrifuged at 15 000 × g, 15 min, 4°C to clear unbroken cells and large debris, followed by ultracentrifugation at 185 511 × *g*, 1 h 15 min, 4°C to collect a membrane fraction containing WzxE. Membranes were solubilized overnight at 4°C in 100 ml of 25 mM Tris pH 7.5, 150 mM NaCl, 5% glycerol (v/v) (buffer A), supplemented with 1.5% (w/v) n-decyl-β-D-maltopyranoside (DM—Glycon). After centrifugation at 185 511 × *g* for 1 h 15 min at 4°C, the supernatant was batch bound onto 3 ml of nickel NTA resin (ABT) equilibrated in PBS for 2 h at 4°C. The beads were then washed in 20 mM and then 35 mM imidazole in buffer B (buffer A supplemented with 0.058% (v/v) octyl glucose neopentyl glycol (OGNG—Anatrace) and 0.008% (v/v) n-dodecyl-β-D-maltopyranoside (DDM—Glycon)). The protein was eluted in buffer B containing 300 mM imidazole and desalted using a CentriPure P100 desalting column (Generon) in buffer B containing 20 mM imidazole. After tag removal by overnight incubation with TEV protease, the protein was further purified by reverse immobilized metal affinity chromatography (ABT beads) and size exclusion chromatography on a HiLoad 16/600 Superose 6 (Cytiva) in buffer B. Fractions containing WzxE were pooled and concentrated using a 100 kDa MWCO spin-concentrator (Sartorius). For the protein purified in DDM only, the same protocol was followed using 1% (w/v) DDM for extraction and 0.024% (v/v) DDM for purification instead. Stain-free Bio-rad SDS-PAGE gels (Any kDa) were run at each step of the purification following the manufacturer’s protocol in Tris-Glycine-SDS buffer, with Precision Plus Protein Unstained standard (Bio-rad).

### 2.3. Nanobody production, expression and purification

To generate a nanobody library, 1 mg (at 1 mg ml^−1^) of WzxE protein sample purified in DM, DDM and OGNG was sent to the Nanobody4Instruct facility (https://www.instruct-eric.org/) in Brussels for llama immunization. Immunizations, library construction, selections and screenings were performed as described before [50]. The process yielded 16 different nanobody clones, each contained within the plasmid pMESy4. Those coded CA12482 and CA12485 were designated Nb7 and Nb10 respectively in this manuscript. The same site-directed mutagenesis method described above [45] was used to exchange the C-terminal hexa-histidine tag of Nb10 in the pMESy4 vector for a strep II tag. Plasmid sequences were confirmed by DNA sequencing.

Nanobody expression and purification were done according to published protocols [50]. Briefly, each plasmid was transformed into WK6 (*su^-^*) *E. coli* cells and grown at 37°C in terrific broth (Melford) to an OD_600nm_ of 0.8. Protein expression was induced with 1 mM isopropyl ß-D-1-thiogalactopyranoside (IPTG) for 16 h at 28°C. The cell pellet was resuspended (30 ml  l^−1^ of culture) in TES buffer (0.2 mM Tris pH 8, 0.5 mM EDTA and 0.5 M sucrose) and 25 µg ml^−1^ DNAse I under agitation for 16 h at 4°C. A further 30 ml l^−1^ of culture of four-fold diluted TES buffer was added with 25 µg ml^−1^ of DNase I before centrifugation for 30 min at 9793 × *g* at 4°C. The filtered supernatant was loaded onto a 5 ml of His-Trap FF column (Cytiva) at 4°C using an ÄKTA PURE system (Cytiva). The column was washed in PBS pH 7.4, 30 mM imidazole. The protein eluted in 10 ml of PBS pH 7.4, 300 mM imidazole, and directly injected onto a gel filtration HiLoad 16/600 Superdex 75 column (Cytiva) in PBS using the peak-to-loop method (Cytiva). The protein fractions were pooled and concentrated using a 5 kDa MWCO spin-concentrator (Sartorius) to 15–20 mg ml^−1^ and stored at −80°C after flash-freezing. The Nb10-strep II tagged protein was expressed and purified as described above, but with the following exception: 3 ml of Strep-tactin beads (IBA) were used, the protein was bound to the resin for 2 h at 4°C and the protein was eluted with 5 mM desthiobiotin in PBS pH 7.4. The protein was then concentrated and eluted on HiLoad 16/600 Superdex 75 column in PBS, pH 7.4.

### 2.4. Synthesis and preparation of nerol-PP-GlcNAc and und-PP-GlcNAc

The syntheses of nerol-PP-GlcNAc and und-PP-GlcNAc are described in electronic supplementary material. Und-PP-GlcNAc (approx. 1 mg) was resuspended in 200 µl of 9:1 chloroform:methanol. The solvent was evaporated using a nitrogen stream and dried under vacuum. The dried lipid film was resuspended at a final concentration of 10 mM in 25 mM Tris pH 7.5, 150 mM NaCl, 0.104% (v/v) DDM and 0.754% (v/v) OGNG. The resulting clear solution was stored at −20°C. For supplementing monoolein with different lipids, und-PP-GlcNAc (approx. 1 mg) and *E. coli* Polar Lipids (ECP, Avanti) (approx. 15–25 mg) were individually resuspended in chloroform and the solvent was evaporated as described above. Monoolein (Molecular Dimensions) was melted at 42°C, added to the ECP to obtain an ECP concentration of 15% (w/v). The mixture was resuspended at 42°C until the compounds dissolved. The monoolein:ECP mixture, warmed at 42°C, was then transferred into a silica glass tube containing the und-PP-GlcNAc at a lipid concentration of 10 mM. The monoolein:ECP:und-PP-GlcNAc solution and the monoolein:ECP were each aliquoted and stored at −20°C.

### 2.5. Structural biology

WzxE protein was incubated with each nanobody at a molar ratio 1:1.2 WzxE:Nb. After binding for 2 h at 4°C, complex formation was assessed by co-elution on size exclusion chromatography in buffer B. The complex WzxE-Nb10 peak fractions were concentrated to 10 mg ml^−1^ with a 100 kDa MWCO spin-concentrator (Sartorius). All protein mixtures were freshly prepared and were centrifuged at 20 798 × *g*, 10 min at 4°C before crystallization. The protein complexes were crystallized in lipidic cubic phase (LCP) at 20°C with the monoolein:ECP (2:3 ratio protein to monoolein) solution in 0.1M Na acetate pH 6.4–6.5, 28–29% (v/v) PEG400, 0.17–0.19 M lithium sulfate, 0.02M zinc chloride for S-SAD or 100 mM Na acetate pH 6.43, 30–31% (v/v) PEG400, 0.18 M lithium sulfate, 0.02 M zinc chloride for native structure, optimized from the LCP screen (Jena Bioscience). Drops were made from 50 nL protein and 850 nL precipitant. Crystals were harvested after two months and were flash cooled in liquid nitrogen using 0.02 to 0.04 mm litho-loops (Molecular Dimensions) for the I24 beamline (Diamond Light Source) or special 0.06 mm litholoops glued to copper pins for the I23 beamline (Diamond Light Source) [51].

The WzxE-Nb10-Nb7 complex was made by adding a 1.2-fold molar excess of Nb7 to the pre-formed WzxE-Nb10 for 1 h at 4°C. Und-PP-GlcNAc (2 mM) was added to the protein complex. Sonication (5 s) and incubation at 4°C for 16 h, was followed by another sonication step (5 s). LCP plates were set up with the monoolein:ECP:und-PP-GlcNAc solution. The plates were incubated at 20°C and crystals obtained in 0.1 M ammonium sulfate, 0.1 M glycine pH 3.8, 28% (v/v) triethylene glycol (TEG) from the Memgold screen (Molecular Dimensions) for the inward-facing conformation and in 0.1 M sodium citrate pH 5, 0.1 M lithium sulfate, 0.1 M sodium chloride, 30% (v/v) PEG300 from the MemMeso screen (Molecular Dimensions) for the outward-facing conformation. Crystallization with nerol-PP-GlcNAc followed a similar protocol using monoolein:ECP mixture but with 10 mM final concentration added to the protein for 16 h at 4°C and without sonication. The inward-facing conformation was crystallized in 0.1 M tri-sodium citrate pH 5, 0.05 M lithium sulfate, 0.1 M sodium chloride and 18.5% (v/v) PEG400 from the LCP Screen (Jena Bioscience). The outward-facing conformation was crystallized in 0.1 M tri-sodium citrate pH 5, 0.37 M ammonium acetate, 3% (v/v) 1-propanol and 35% (v/v) PEG400 from the LCP Screen (Jena Bioscience).

All diffraction data were collected at Diamond Light Source. The lack of consistent crystal formation and diffraction reproducibility meant that only approximately 15% of the tested crystals diffracted beyond 3 Å, requiring extensive pre-screening using the I03 beamline. To maximize the anomalous diffraction arising from sulfur atoms, data were collected on the I23 beamline at a wavelength of 2.7552 Å using 0.1 s exposure with 0.1° oscillation and 50% transmission. With the multi-axis goniometer, multiple datasets from the same crystal were collected at different kappa angles. In total, 21 datasets from four different crystals were collected, and the data were integrated with XDS and merged using XSCALE from the XDS suite (RRID:SCR_015652 [52]). The structure was solved by SAD phasing. The experimental phased map was calculated by CRANK2 [53] from the CCP4 suite (RRID:SCR_007255 [54]). The nanobody was manually fitted and Autobuild in Autosol [55] from the Phenix suite (RRID:SCR_014224 [56]) and Buccaneer (CCP4 [57]) were used to build the structure. The data (PDB-ID: 9G95) was deposited to the PBD (RRID:SCR_012820). A native dataset was collected at 2.3 Å on the I24 beamline at a wavelength of 0.9688 Å at 20% transmission with 0.02 s exposure, 0.1° oscillation and a beam size of 20 × 20 µm with a Pilatus 6M detector. The data were deposited to the PBD (PDB-ID: 9G97).

The WzxE-Nb7-Nb10 complex data were also collected between 2.55 to 2.8 Å on I24 using a wavelength of 0.6199 Å on the CdTe Eiger2 9M detector with the same previous settings except a beam size of 7 × 7 µm and a transmission of 50%. The data was auto processed by the Diamond Light Source pipeline using Xia2 for integration [58]. The I24 data was solved by molecular replacement using Phaser (CCP4) [59] and the structure was refined by a combination of Phenix.refine (Phenix) and Refmac (CCP4) [60]. The quality of the structures was checked with MolProbity (RRID:SCR_014226 [61]). The data were deposited to the PDB (PDB-IDs: 9G9M, 9G9N, 9G9O, 9G9P). The structure figures were produced with Chimera (RRID:SCR_004097 [62]) and Pymol (RRID:SCR_000305). The electrostatic potential of the WzxE structures was calculated using the QtMG program of the CCP4 program suite.

### 2.6. Enzyme-linked immunosorbent assay

The Nb10-Strep II tagged protein was biotinylated using the EZ-link Sulfo-NHS-LC Biotin kit (Thermofisher Scientific), following the manufacturer’s protocol. Excess biotin was removed by two dialysis steps in 1 l PBS, 4°C, overnight. The enzyme-linked immunosorbent assay (ELISA) was performed at room temperature, with three replicate wells for each condition. Streptactin-coated 96-well plates (Pierce) were washed in 25 mM Tris pH 7.5, 150 mM NaCl, 0.02% (v/v) DDM, 0.1% (w/v) BSA and 0.05% (v/v) Tween-20 (buffer C). Biotinylated Nb10-strep II tagged protein at 1.62 µM/well in buffer C was bound for 2 h. The plate was washed and blocked in buffer C using 0.5% (w/v) BSA for 30 min. The plate was washed, then WzxE purified in DDM was added to the plate at a concentration of 2.78 µM and incubated for 1 h. After washing, the anti-His HRP antibody (Sigma-Aldrich, A7058, RRID:AB_258326) was added to the plate at a 1:2000 dilution in buffer C. The plates were washed and incubated with the substrate solution A:B of Sera Care KPL Peroxidase substrate (Sera Care 5120-0037 and 5120-0034) and the signal was acquired using a plate reader (Clariostar—BMG Labtech) at 410 nm after 30 min incubation. The values were plotted using Prism 10 (GraphPad, RRID:SCR_002798).

### 2.7. Analysis of the ECA phenotype in mutants by immunoblotting

CWG1573 containing pBAD24 derivatives encoding wild-type or mutant *wzxE* together with *wecF*, *wzyE* and *wecG*, were grown for 16 h in LB at 37°C. One OD_600nm_ unit of cells was collected by centrifugation at 12,000 × g for 1 min and the pellet was lysed in 100 µl of SDS-PAGE loading buffer (62.5 mM Tris-Cl pH 6.8, 2% (w/v) SDS, 10% glycerol, 5% (v/v) β-mercaptoethanol, 0.002% (w/v) bromophenol blue). CWG1573 transformants containing mutations in *wzxE* affecting any of the C-terminal arginine residues (R413, R414, R415) would not grow in liquid media but would grow on LB agar. Cultures of these were scraped from agar plates, resuspended in water and 1 OD_600nm_ unit of cells from the suspensions were collected and lysed as described for the liquid cultures. Whole-cell lysates were heated in a boiling water bath for 10 min and then treated with 0.5 mg ml^−1^ proteinase K for 30 min at 55°C. Samples (10 µl) were separated by SDS-PAGE in Tris-glycine buffer [63] on 12% gels at 150 V for 1 h. After electrophoresis, samples were transferred to nitrocellulose membrane (Amersham Protran 0.45 µm NC, Cytiva) in 25 mM Tris, 150 mM glycine, 20% (v/v) methanol at 200 mA for 45 min. The immunoblots were blocked in 5% (w/v) skimmed milk, probed with ECA-specific rabbit antiserum and alkaline phosphatase-conjugated goat anti-rabbit secondary antibody (Cedarlane Laboratories). Blots were developed using nitroblue tetrazolium and 5-bromo-4-chloro-3-indolyl phosphate (Roche Applied Science) substrates.

### 2.8. WzxE sequence alignment and cavity volume calculations

A set of 1995 WzxE sequences from the Uniprot database [64] LRRID:SCR_002380) were aligned using the Clustal Omega server [65] (RRID:SCR_001591). A consensus sequence was derived using the Jalview program [66] (RRID:SCR_006459). A smaller set of 10 representative WzxE sequences from different *Enterobacterales* having an AlphaFold model (RRID:SCR_023662) were obtained from the Uniprot database from the following organism: *E. coli*, *S. typhimurium*, *H. alvei*, *P. mirabilis*, *K. pneumoniae*, *C. freundii*, *E. hormaechei*, *S. marcescens*, *M. morganii* and *S. flexneri*. Those sequences were aligned with ESPript 3.0 [67]. The structures were aligned using Pymol.

## 3. Results

### 3.1. The WzxE-Nb10 structure is in an outward-facing conformation

**Figure 2.**
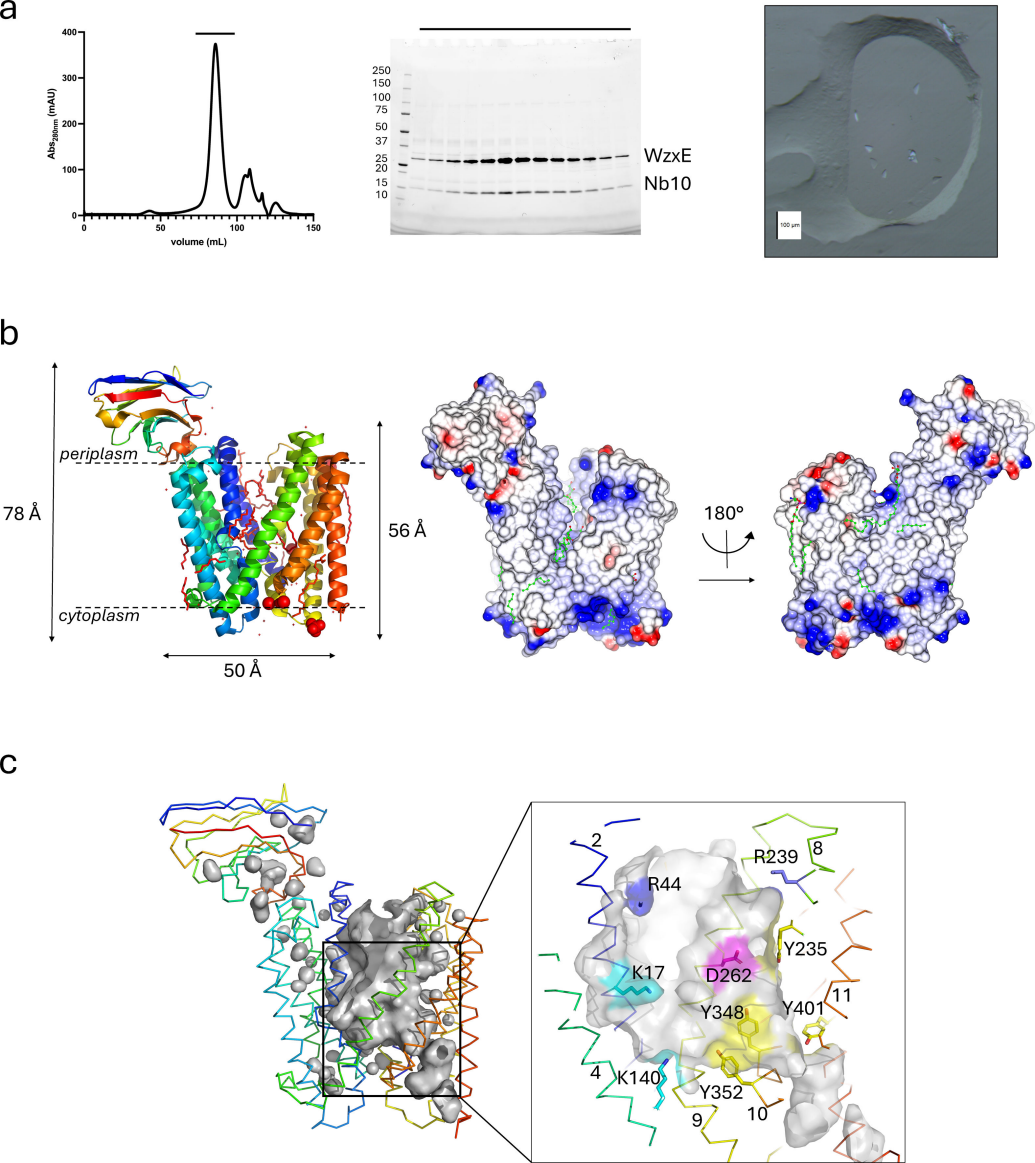
WzxE biochemistry and outward-facing X-ray structure. (*a*) Size exclusion chromatography of the WzxE-Nb10 purified complex. The elution chromatogram is shown in the left panel; the corresponding stain-free SDS-PAGE gel with the fractions highlighted is shown in the middle panel. A picture of the typical protein crystals obtained after two months are shown on the right panel. (*b*) X-ray structure of the original WzxE-Nb10 complex (PDB-ID: 9G97) was solved by molecular replacement using the S-SAD I23 structure (PDB-ID: 9G95). Nb10 binds the N-terminal lobe of the protein. The structure is represented using chainbow colouring; with the ligands shown in magenta (left panel). The electrostatic potential of the WzxE structure is represented in surface mode (middle panel), with the ligands in green. A 180° rotation using the vertical axis of the same representation is also shown (right panel). (*c*) The WzxE internal cavity for the original outward-facing structure is represented in grey surface mode, with the protein chains represented in ribbon mode in chainbow colours, for the original outward-facing structure (PDB-ID: 9Q97—left panel). The insert right panel shows a zoomed representation of the internal pocket of the outward-facing structure. Important residues are shown in sticks mode, in blue for arginine, magenta for glutamate, cyan for lysine and yellow for tyrosine residues. The helix numbering is also shown.

After extensive trials, only WzxE in a combination of detergents (DM for extraction; DDM/OGNG for purification) in complex with nanobody Nb10 was crystallized in lipid cubic phase (figure 2*a*; electronic supplementary material, S3b). The initial crystal hit appeared after a week and the crystals reached their full size (approx. 10 µm) after approximately 2–3 months at 20°C. The addition of zinc chloride to the precipitant and *E. coli* polar lipids to the monoolein at a concentration of 15% resulted in larger and more reproducible diffracting crystals. Both molecular replacement with homologous structures (prior to the AlphaFold model) and experimental phasing using selenomethionine labelling of WzxE and/or Nb10 failed. The first map of the WzxE-Nb10 complex was obtained using sulfur phasing and a low-resolution model was built. This model was used to obtain a higher-resolution map and model using the I24 dataset (table 2).

**Table 2.** X-ray crystallography data collection and refinement. Data were collected from four crystals for the 9G95 structure and from a single crystal for the other structures. Values in parentheses are for highest-resolution shell.

	WzxE-Nb10		WzxE-Nb10-Nb7_out_		WzxE-Nb10-Nb7_in_	
	I23 S-SAD	I24 native	crystal 1	crystal 2	crystal 1	crystal 2
PDB-ID	9G95	9G97	9G9M	9G9O	9G9N	9G9P
**data collection**						
wavelength (Å)	2.755	0.9688	0.6199	0.6702	0.6199	0.6702
space group	*C*2	*C*2	*C*222_1_	*C*222_1_	*C*222_1_	*C*222_1_
cell dimensions						
a, b, c (Å)	271.54 38.45 56.96	270.55 38.47 56.83	38.85 152.68 287.50	84.24 191.91 101.41	42.34 154.02 288.08	83.8 191.68 102.09
α, β, γ (°)	90.00 93.09 90.00	90.00 93.50 90.00	90.00 90.00 90.00	90.00 90.00 90.00	90.00 90.00 90.00	90.00 90.00 90.00
resolution range (Å)	45.19–2.80 (2.95–2.80)	51.19–2.31 (2.40–2.31)	32.52–2.55 (2.64–2.55)	38.50–2.6 (2.80–2.69)	32.40–2.80 (2.95–2.80)	34.94–2.80 (2.95–2.80)
unique reflections	14 932 (2147)	25 882 (2855)	28 333 (2547)	26 804 (2868)	20 563 (2871)	20 501 (2839)
completeness (%)	100.00 (99.90)	98.67 (98.11)	98.75 (90.58)	99.30 (97.62)	99.67 (99.83)	99.68 (98.85)
multiplicity	85.10 (19.60)	6.60 (6.40)	6.20 (4.10)	6.80 (6.90)	6.70 (6.90)	6.80 (7.00)
Wilson B-factor (Å^2^)	66.45	42.56	47.64	55.56	57.79	49.65
R-meas (%)	15.90 (105.60)	18.20 (173.10)	26.20 (213.10)	39.00 (328.70)	28.00 (180.30)	21.40 (108.60)
CC1/2	0.99 (0.87)	1.00 (0.40)	1.00 (0.30)	1.00 (0.30)	1.00 (0.70)	1.00 (0.80)
anomalous						
completeness (%)	100.00 (99.90)					
multiplicity	43.90 (9.90)					
anomalous slope	1.15					
**refinement**						
resolution range (Å)	44.78–2.80 (3.02–2.80)	51.19–2.31 (2.40–2.31)	32.52–2.55 (2.64–2.55)	38.50–2.69 (2.8–2.69)	32.4–2.8 (2.95–2.8)	34.94–2.80 (2.95–2.80)
reflections used in refinement	14 921 (2933)	25 882 (2855)	28 333 (2547)	26 804 (2868)	20 563 (2871)	20 501 (2839)
R-work (%)	23.76 (29.05)	21.40 (27.55)	23.45 (33.80)	27.99 (38.29)	25.57 (36.37)	22.01 (27.35)
R-free (%)	29.14 (36.44)	26.08 (34.59)	28.68 (39.33)	33.86 (39.21)	31.34 (44.04)	27.33 (33.58)
no. of atoms						
protein	4131	4131	5094	5101	5060	5045
non-hydrogen atoms	4309	4450	5137	5101	5060	5045
ligand	178	274	5	0	0	0
water	0	45	38	0	0	0
protein residues	543	543	666	668	662	660
RMSD						
RMS (bonds)	0.003	0.005	0.002	0.002	0.002	0.002
RMS (angles)	0.66	0.76	0.41	0.46	0.51	0.46
Ramachandran						
favoured (%)	98.52	98.33	96.82	97.43	96.49	96.79
allowed (%)	1.48	1.30	3.18	2.42	3.51	3.21
outliers (%)	0.00	0.37	0.00	0.15	0.00	0.00
rotamer outliers (%)	0.95	0.95	1.53	0.77	1.35	1.35
clashscore	5.53	5.49	2.72	6.99	9.80	5.02
B-factor						
average B-factor	64.43	51.63	57.13	57.36	68.98	59.01
macromolecules	63.94	50.99	57.17	57.36	68.98	59.01
ligands	75.87	61.92	69.75			
solvent		47.59	49.79			

WzxE (named chain A in the PDB model) crystallizes as a monomer (figure 2*a,b*) with the Nb10 nanobody on the periplasmic face. The original WzxE-Nb10 structure (PDB-ID: 9G97) revealed a topology of a N-terminal and a C-terminal domain (figures 1*c* and 2b) each consisting of a six α-helical bundle (residues 1–210 of transmembrane (TM) helices 1–6 and 211–418 of TM 7–12) separated by an internal portal open towards the periplasm [35]. TM1 (residues 1–32) and 7 (residues 211–245) from one side of the bundle extend into the other bundle (figures 1*c* and 2*b*). The N- and C-terminal residues are both on the same membrane side (figures 1*c* and 2*b*), confirming earlier biochemical studies that annotated them as both cytoplasmic [68]. Despite sequence similarity as low as 4.6% with the consensus sequence from 52 representative MATE_like proteins, the WzxE protein structure clearly identifies it as a member of the MATE family [41]. As predicted [32], WzxE shows high similarity to NorM-Vc (PDB-ID: 3MKT [35]) with an RMSD of 1.2 Å over 533 residues. One interesting feature of WzxE is the arrangement of TM3 and TM9 (figures 1*c* and 2*b*) resulting in a kink at the end of each helix which creates a small α-helix running parallel to the membrane of the periplasmic face. The distortion in the helix is due to the presence of a proline P104 in TM3 and the presence of bulky hydrophobic amino acids within TM9, such as W312, W320 and F317. In the N-terminal domain, three tryptophan residues (W9, W131, W209) sit just above the cytoplasmic face. In the C-terminal bundle, a ring of exposed tryptophan residues (W312, W336, W380) sits on the outer surface of the structure just below the periplasmic face. Similar arrangements of exposed tryptophan residues are commonly found in transmembrane proteins, where they serve as anchors by sitting in (but close to the surface of) the hydrophobic region [69]. WzxE binds a single nanobody molecule (Nb10—chain B) on the periplasmic side (figure 2*b*). The complementarity-determining region (CDR) 3 loop (loop 8–9) of the Nb10 binds the WzxE loops 1–2, 3–4 and 5–6 of the N-terminal domain. The crystal contacts were mediated mostly by the interaction of the cytoplasmic hydrophilic face of WzxE and between the nanobody on the periplasmic face.

### 3.2. The WzxE-Nb10-Nb7 complex crystallizes in both inward and outward-facing conformations

**Figure 3.**
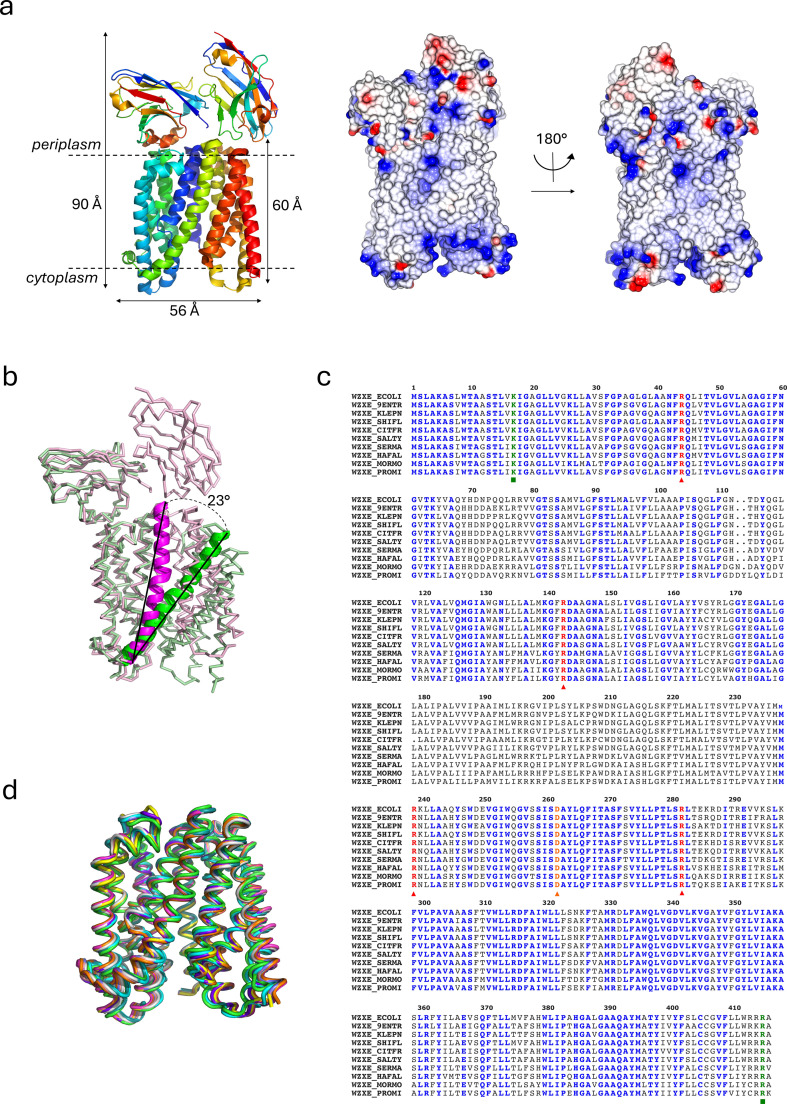
WzxE inward-facing structure and comparison with other WzxE AlphaFold structures. (*a*) X-ray structure of the WzxE-Nb10-Nb7 complex in inward-facing conformation (PDB-ID: 9G9N) is shown in the same representation mode as figure 2*b*. (*b*) Superposition of N-lobe of the outward-facing (9G97 in green) and inward-facing WzxE (9G9N in magenta). The protein is represented in ribbon mode with the TM7 in cartoon mode. The angle shift between outward-facing and inward-facing was calculated using coot from the CCP4 suite. (*c*) Alignment of a set of 10 WzxE sequences from different *Enterobacterales*, with amino-acid numbering from the WzxE *E. coli* sequence. The conserved residues are in highlighted in different colours. (*d*) Structural alignment of the 10 WzxE AlphaFold models for the sequences represented in (*c*): 9G97 (green), *S. typhimurium* (cyan), *H. alvei* (magenta), *P. mirabilis* (yellow), *K. pneumoniae* (salmon), *C. freundii* (grey), *E. hormaechei* (slate), *S. marcescens* (orange), *M. morganii* (purple) and *S. flexneri* (teal). The helices are represented in cartoon tube mode.

Nanobodies recognizing a different binding site to Nb10 were sought to obtain a different conformation of WzxE. These were screened using an ELISA assay in the presence of the WzxE-Nb10 complex. Four nanobodies from different families were found to bind the WzxE-Nb10 complex, implying a binding site not occluded by Nb10. Nb7 gave the strongest signal, suggesting strongest binding (electronic supplementary material, figure S2a). Nb7 was therefore purified (electronic supplementary material, figure S3a) and added to WzxE but no diffracting crystals were obtained for the WzxE-Nb7 complex (electronic supplementary material, figure S4a). Crystals were only obtained with a combination of Nb10 and Nb7 (figure 3*a*; electronic supplementary material, S2b–d and S4c). WzxE-Nb10-Nb7 complexes were crystallized in presence of polyprenol-PP-GlcNAc substrates, und-PP-GlcNAc (PDB-ID: 9G9O and 9G9P) or nerol-PP-GlcNAc (PDB-ID: 9G9M and 9G9N) although neither substrate was observed in the electron density that were obtained for both conditions (electronic supplementary material, figures S16 and S11). Other nanobodies tested either individually, or as combinations, did not yield diffracting crystals in our hands.

The WzxE-Nb10-Nb7_out_ structures were solved by molecular replacement using the WzxE-Nb10 structure as template, refined to a resolution of 2.55 Å (PDB-ID: 9G9M) and 2.69 Å (PDB-ID: 9G9O) (table 2). The WzxE N-terminal lobe with the nanobody Nb10 superpose well with the original WzxE-Nb10 structures, suggesting a similar binding of Nb10 to WzxE in the presence of Nb7 (electronic supplementary material, figure S2b,c). Extra density can be obtained corresponding to the Nb7 (chain C), linked to the C-lobe on the periplasmic side (electronic supplementary material, figure S2b,c). The Nb7 binding is similar between the two structures. The three CDRs of Nb7 (loops 2–3, 4–5 and 8–9) interact with the loops 7–8, 9–10 and 11–12 of WzxE. Despite the addition of Nb7, the structures superpose well with the original structure (RMSD 1.16–1.22 Å over 532–531 residues), with a slight approximately 3° shift of the C-terminal helical bundle of WzxE relative to the original structure starting from residue 221, suggesting that both structures are in the outward-facing conformation.

Additional crystallization conditions were identified for the WzxE-Nb10-Nb7 complex. Crystals of WzxE-Nb10-Nb7_in_ diffracted to 2.8 Å (PDB-ID: 9G9N and 9G9P; table 2) showing an essentially identical structure. The structure has an occluded inward conformation, i.e. open towards the cytoplasm (figure 3*a*; electronic supplementary material, S2d). This arrangement superposes well with the subsequent WzxE AlphaFold model (RMSD 1.11 Å over 410 residues; AF-P0AAA7-F1) and also with other WzxE structures predicted by AlphaFold from 10 different *Enterobacterales* (RMSD 1.06 to 2.15 Å over 408 residues) (table 3; figure 3*c,d*). Each helical bundle superimposes closely with their counterparts in the other WzxE *E. coli* structures (maximum RMSD 0.76 Å over 208 residues for the N-terminal bundle and maximum RMSD 1.62 Å over 168 residues for the C-terminal bundle) with their counterparts in the other WzxE *E. coli* structure, however the overall structures do not superpose well (maximum RSMD 4.56 Å over 360 residues), as the arrangement of the two helical bundles differs (figure 3*b*).

**Table 3.** Cavity volume of different WzxE and Wzx O-antigen flippase protein structures calculated with CASTp 3.0. Branched sugars are written in square brackets.

Protein	Strain	PDB-ID or AlphaFold-ID	Number of sugar substrate	Polysaccharides	CASTp area (Å^2^)	CASTp volume (Å^3^)	Reference
WzxE	*E. coli*	9G97 (outward)	3	Lipid III: >3)-α-d-FucNAc4-(1>4)-β-d-ManNAcA-(1>4)-d-GlcNAc-(1>	2578.596	3154.112	this study
WzxE	*E. coli*	9G9M (outward)	3		2390.816	3059.370	this study
WzxE	*E. coli*	9G9N (inward)	3		2536.334	2575.829	this study
WzxE	*E. coli*	9G9O (outward)	3		2589.218	3629.924	this study
WzxE	*E. coli*	9G9P (inward)	3		2328.101	2409.121	this study
WzxE	*E. coli*	AF-P0AAA7-F1	3		2557.213	2623.837	this study
WzxE	*S. flexneri*	AF-P0AAA8-F1	3		2463.832	2261.163	[70]
Wzx O157	*E. coli*	AF-Q7DBF2-F1	4	O157: >2)-α-d-PerNAc-(1>3)-α-l-Fuc-(1>4)-β-d-Glc-(1>3)-α-d-Gal2NAc-(1>	3563.162	5360.612	[23]
WzxC	*E. coli*	AF-P77377-F1	5	Colanic acid: >3)-[4,6-pyr-β-d-Gal-(1->3)-β-d-GlcA-(1>3)-β-d-Gal-(1>4)]-β-l-Fuc-(1>4)-3-OAc-α-l-Fuc-(1>3)-β-d-Glc-(1>	3815.283	5707.812	[71]
Wzx	*P. aeruginosa*	AF-G3XD19-F1	3	O5: >4)-d-ManNAc3NAmA-(β1>4)-d-ManNAc3NAcA-(β1>3)-d-FucNAc-(α1>	2587.784	5589.980	[68,72,73]
Wzx	*P. aeruginosa*	AF-Q9XC64-F1	3	O11: >3)-d-Glc-(β1>3)-l-FucNAc-(α1>3)-d-FucNAc-(β1>	2115.654	2628.642	[72,73]
Wzx	*P. aeruginosa*	AF-Q9RHD4-F1	4	O6: >3)-l-Rha-(α1>4)-d-GalNAcA3Ac-(α1>4)-d-GalNFoA-(α1>3)-d-QuiNAc-(α1>	2102.105	3035.326	[72,73]
Wzx	*S. enterica*	AF-S5R5C7-F1	4	O9: >6)-[α-d-tyv-(1>3)]-β-d-Man-(1>4)-α-l-Rha-(1>3)-α-d-Gal-(1>	2077.800	3585.001	[74]
Wzx	*E. coli*	AF-Q9X4C7-F1	4	K30: >2)-[β-d-Glc*p*A-(1>3)-α-d-Gal*p*-(1>3)]-α-d-Man*p*-(1>3)-β-d-Gal*p*-(1>	2836.825	4002.582	[75]

The change of conformation between outward-facing and inward-facing is mediated by the movement of the TM7 which rotates approximately 23°, starting at residues N211 (figure 3*b*). The rotation of the two protein lobes is facilitated by the bending of TM1 starting from G19 (figure 1*c*). In each structure, the WzxE residues V52 to I58 in TM2 are disordered indicative of multiple conformations. A small loop is created in TM2 due to the presence of glycine and alanine residues (_54_AGAG_57_) which bends towards the inside of the helical bundle, creating a larger internal cavity. Interestingly, the WzxE AlphaFold structure indicates this region (G55 to I58) is disordered. While the interaction of WzxE with Nb10 and with Nb7 is similar to the one in the WzxE-Nb10-Nb7_out_ structure (figure 3*a*; electronic supplementary material, S2b,c), this new conformation brings the two nanobodies close enough to interact with each other. The N-terminus and CDR1 of Nb10 contacts the edges of the β-sheets of Nb7; these interactions would appear to preclude further inward movement.

Additional densities were observed in the different maps, but these were inconsistent with pyrophosphate groups and were located outside the central cavity; we concluded they were not bound substrates. Instead, they were more convincingly assigned as potential associated lipids (monoolein or others) (figure 2*b*; electronic supplementary material, S1a). A sulfate (or a phosphate) ion from the crystallization condition (or buffer) is located adjacent to R415, a second ion is coordinated by R331, and a zinc chloride molecule is found close to the first sulfate and the stretch of arginine residues in the C-terminal tail (electronic supplementary material, figures S1b,c and 4*a*).

**Figure 4.**
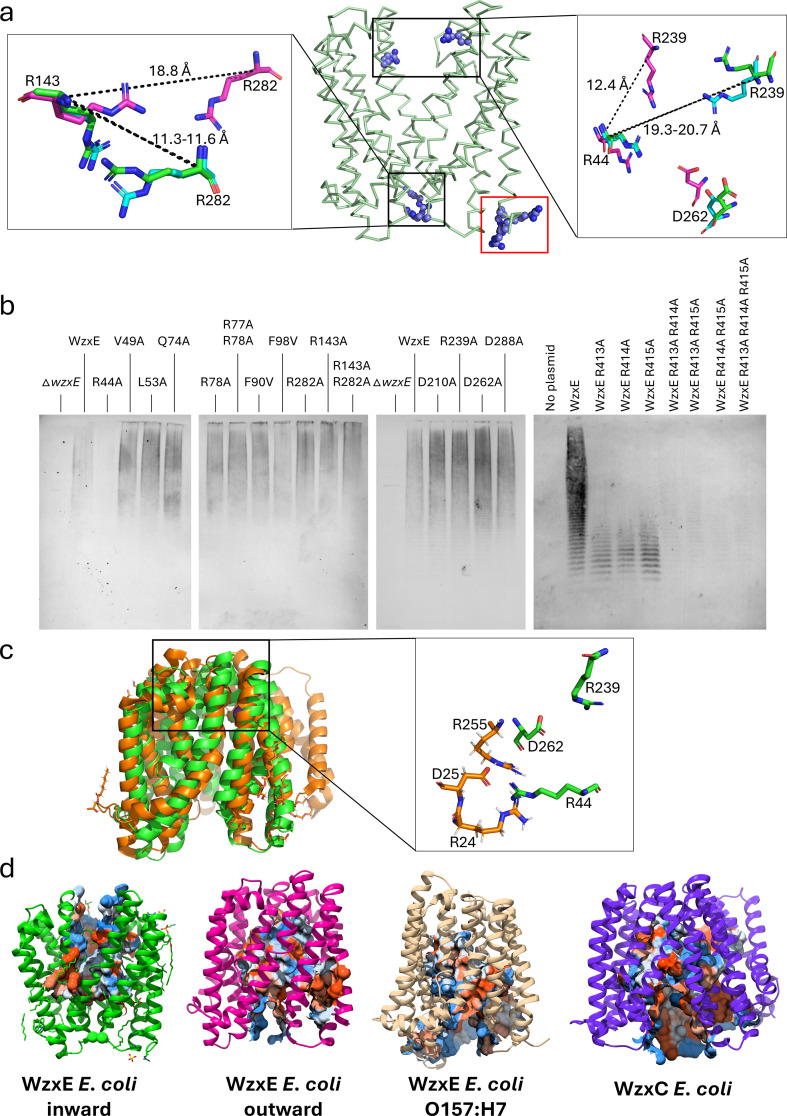
Activity of the WzxE flippase. (*a*) Critical arginine residues R44, R143, R239, R282, R413−415 and their positions in WzxE are shown. A zoomed-in representation of the arginine pairs is shown in stick mode in the left (R143–R282) and right (R44-R239-D262) inserts. Residues in the original outward-facing structure are coloured in green, the outward-facing WzxE-Nb10-Nb7 structure in blue and the inward-facing WzxE-Nb10-Nb7 structure in magenta. The distances between the Cα of the arginine residue pairs for each structure are shown. The red rectangle highlights the stretch of C-terminal arginine residues stretch (R413−415). (*b*) Western blot of ECA production in different mutant strains. ECA production is detected using an anti-ECA antibody. (*c*) Superposition of the WzxE-Nb10-Nb7 inward-facing structure (PDB-ID: 9G9N) without nanobodies in green and of the lipid II flippase MurJ (PDB-ID: 6NC8) in inward occluded conformation in orange. A zoom insert of the catalytic triad (R24-D25-R255) of MurJ is shown in orange, while the WzxE counterpart is shown in green. (*d*) Inner cavity of WzxE and Wzx computed by CASTp 3.0 [76]. The original outward-facing is represented in green and the inward-facing in magenta; AlphaFold models of WzxE *E coli* O157:H7 (AF-Q7DBF2-F1) and WzxC *E. coli* (AF-P77377-F1) are shown in beige and purple, respectively.

### 3.3. Structural analysis of the WzxE central cavity

The central cavity of WzxE is mostly formed by TM1−2 and TM7−8 and part of TM4 and TM10 (figures 1*c* and figure 2*c*). The stretch of arginine residues (R413−415) on the C-terminal tail are close to a sulfate molecule (figure 4*a*; electronic supplementary material, S1b), and is positioned adjacent to the cytoplasmic entrance to the central lumen (figures 1*c*, 2c and 4*a*). As predicted by a tertiary structural homology model for Wzx_Pa_ [32], the cytoplasmic (K64, R143, R282) and the periplasmic (R44, R239, K326) parts of the lumen are dominated by positively charged residues. The side chains of residues K17, R44, R143 and R282 point into the middle of the cavity (figure 2*c*).

Comparing the structures of different WzxE conformations, the cytoplasmic pair R143–R282 are separated by approximately 12 Å (Cα to Cα) in the outward configurations but 19 Å in the inward structure (figure 4*a*). The periplasmic R44–R239 pair is separated by approximately 19 Å (Cα to Cα) in the outward configurations and 12 Å in the inward conformation. The inverse relationship between the pairs of arginine residues at either side of the centre of the lumen is striking. Both arginine residue pairs also have adjacent lysine residues. The periplasmic R44–R239 pair are close to the partially conserved aspartic acid D262 (figure 4*a*), and a similar arrangement is seen in MurJ, where the three residues form a salt bridge [24]. We did not observe a comparable salt bridge in the WzxE structures but conformational changes in the side chains could plausibly create one. The carboxylate side chain of D262 would be available to coordinate any metal ion bound to the pyrophosphate of lipid III or to the sugar hydroxyls. At the cytoplasmic face, a number of glutamate residues surround the entrance to the lumen but none in the same position relative to the R143–R282 as seen for D262, R44 and R239. We also note another cluster of positively charged residues R315 and R331, as previously proposed [32].

A sequence alignment of 10 WzxE sequences for which AlphaFold models were established, sharing a UniProt code, show that those four arginine and one aspartate residues are indeed conserved (figure 3*c*). However, a larger Clustal Omega alignment of 1995 Uniprot WzxE sequences from *Enterobacterales* was used to derive a consensus sequence, which only has 32.9% identity with the *E. coli* WzxE sequence used in this work. This low percentage of identity within these WzxE proteins but also more generally Wzx, makes it more difficult to pinpoint the functionally conserved amino acids. While most highly conserved amino acids are glycine, leucine, proline and alanine residues, the only conserved positively charged amino acid is R239 (100%). The other important amino acids flagged previously have a high degree of conservation (R44: 88%, R143: 88%, D262: 90%, R282: 88%), reinforcing their importance for the translocation function.

### 3.4. WzxE activity assays

Attempts to establish an *in vitro* transport assay with WzxE, either in detergent or inserted into liposomes were unsuccessful. An *in vivo* approach was, therefore, chosen to test the function of WzxE mutant derivatives, by complementing the transport defect in a *E. coli* Δ*wzxE* mutant. The *E. coli* K-12 derivative, SΦ874, was chosen as the parental strain for the Δ*wzxE* mutant, as it contains a genomic deletion encompassing the *wzx* flippases involved in colanic acid and O-polysaccharide biosynthesis. The use of this strain was thought to avoid potential functional contributions (crosstalk) to ECA export by Wzx paralogues in a *wzxE* mutant [26]. Although *wzx* mutants have been reported in other *E. coli* K-12 backgrounds containing *wzxE* [26,27,77,78], attempts to delete the *wzxE* open reading frame in SΦ874 were unsuccessful. All colonies growing post-mutagenesis possessed other deletions or secondary (inactivating) mutations in the ECA-biosynthesis operon. This observation may reflect a lethal phenotype caused by sequestration of und-P due to cytoplasmic accumulation of und-PP-linked ECA intermediates [3], when the repeat unit glycosyltransferases are functional in the absence of flippase activity. Defects that accumulate intermediates in the LPS O-antigen, LPS core-oligosaccharide or in the ECA biosynthesis pathways sequester und-P away from the critical peptidoglycan biosynthesis machinery and are known to exert a fitness cost [77,78]. Individual defects within these pathways cause cell envelope and growth defects and have been shown to cause accumulation of secondary suppressor mutations [78]. It is conceivable that mutating *wzxE* in SΦ874, which also lacks the O-antigen and colanic acid flippases, may eliminate diversion of und-P to pathways in which it can be recycled sufficiently to support growth. To mitigate the observed lethality when deleting only *wzxE*, an alternative approach was used in which a genomic deletion spanning the genes *wzxE-wecG* (figure 1*b*) was constructed in SΦ874 (generating *E. coli* CWG1573). The initiating GlcNAc-1-P transferase (WecA) reaction is reversible [79] and the presence of a functional WecA was considered unlikely to affect the viability of *wzxE* mutants in this deletion. ECA biosynthesis was restored by transformation of CWG1573 with a plasmid carrying wild-type *wzxE-wecG* (figure 1*b*). To test the functions of *wzxE* site-directed mutants, each derivative was cloned into a pBAD24 vector contiguously with *wecF-wzyE-wecG* and introduced into *E. coli* CWG1573. Immunoblotting of protease-treated whole-cell lysates was then used to analyse the ECA phenotypes of the *wzxE* mutants. It was hypothesized that this approach would allow identification of any mutants that retained at least some ECA assembly and completely null transport mutants would either not be recovered or could only grow by inactivating ECA synthesis.

Sixteen single amino acid residues were replaced by mutagenesis but most of the alanine mutations had no effect on ECA production (figure 4*b*). The alanine residue mutation of the positively charged R44 residue (R44A) at the lumen of WzxE-Nb10 severely reduced the amount of ECA observed by immunoblot, relative to wild-type WzxE, implying this residue was important for export of the lipid-linked ECA repeat unit. By contrast, the R239 residue that is also exposed in the lumen does not seem to be critical for WzxE activity as the ECA profile of the corresponding R239A mutant was similar to wild type (figure 4*a*,*b*). Although the R44A mutation affected ECA production, only partial loss of ECA export/synthesis was observed; indeed, a null export phenotype would impose lethality in this genetic background. The negatively charged D262 is exposed in the central cavity (figure 4*a*) but a D262A mutant exhibited ECA at a level comparable with wild-type WzxE (figure 4*b*). This result suggests that only R44 is essential for ECA production with the help of R239 and D262. Residue K17 protrudes into the central lumen of WzxE (figure 2*c*) and may interact with the pyrophosphate of the lipid III molecule. The phenotype of a K17A mutant could not be assessed by immunoblotting as we were unable to transform CWG1573 with a plasmid encoding WzxE_K17A_-WecF-WzyE-WecG despite numerous attempts. We interpret this negative result as a result of complete loss of WzxE function in the K17A mutant derivative; however protein toxicity is an alternative explanation. Finally, the levels and sizes of ECA observed in immunoblots were visibly reduced in individual R413A, R414A and R415A mutants (figure 4a,*b*; electronic supplementary material, S1b). When these residues were substituted in pairs or as a triplet, a further reduction of ECA was observed. These data provided evidence for the importance of these terminal arginine residues for WzxE function. The *in vivo* studies show that the residues K17, R44 and R413−415 are critical for the protein function, which is in accordance with the structures, but also with their conservation as shown by the sequence alignment of 10 WzxE AlphaFold models (figure 3*c*).

## 4. Discussion

Despite its relatively low sequence similarity, the structure of WzxE indicates that it is clearly a member of the MATE family [41]. The prototypical MATE family transporter structure, first described in NorM-Vc [35], possesses a central lumen surrounded by two bundles of helices. The outward WzxE-Nb10 structure superimposes with NorM-Vc (RMSD 1.2 Å over 533 residues, PDB-ID: 3MKT [35]), the eukaryotic MATE AtDTX14 (RMSD 2.8 Å over 365 residues, PDB-ID: 5Y50 [80]) and the DinF subfamilies pfMATE (RMSD 2.5 Å over 394 residues, PDB-ID: 3VVN [34]). MATE structures have generally been reported in the outward-facing conformation [81]. However, DinF-BH (PDB-ID: 6FHZ) and PfMATE (PDB-ID: 4LZ6) were solved in an inward-facing conformation [34,35,82]. This PfMATE structure is more ‘open’ than the WzxE-Nb10-Nb7_in_ structure, a finding we attribute to the positions of the bound nanobodies Nb7-Nb10, which would clash with each other in a more open WzxE. The WzxE structure resembles the lipid II flippase MurJ_Ta_ (RMSD 2.913 Å over 351 residues, PDB: 6NC9 [43]) from the peptidoglycan assembly pathway. MurJ was solved in inward-facing conformations with different degree of openness [39,43] and an outward-facing conformation [43]. Comparison of WzxE-Nb10-Nb7 with the MurJ structures lead us to conclude that WzxE-Nb10-Nb7_in_ is an inward occluded form due to the interaction of the nanobodies.

The superposition of individual N- and C-helical bundles in WzxE structures showed that while each bundle is structurally conserved, it is the spatial relationship of the two helical bundles to each other which changes. The conformational change between inward- and outward-facing is mediated by the movement of TM7 which rotates approximately 23° (figure 3*b*). This shift of TM7 between the two bundles was seen in both MurJ [43] and NorM [34]. In the WzxE structures, the disordered region of TM2 (_54_AGAG_57_) could be flexible and play a role in substrate recognition. This region was suggested as functionally important in the studies of Wzx_O157_ and Wzx_Pa_ [32,74,83,84]. A similar stretch of MurJ (G/A-EGA) is present on TM2 and is thought to help the rearrangement of the N-lobe during the change of protein conformation [43]. A small bending of TM8 in WzxE (figures 1*c*, 2b and 3a) was also seen in NorM [34], DinF [33] and MurJ [43]. In MurJ, TM1 and TM2 were also identified as key to conformational changes [43]. Similar large helical bundle movements have been seen for the lipid-linked oligosaccharide PglK flippase [85–87], the lipid A-core OS flippase MsbA [88,89] and the O-antigen ABC transporter Wzm/Wzt [90]. However, the involvement of other helices beyond TM7 are not seen in WzxE, suggesting that only TM7 is essential for the change from an inward- to an outward-facing conformation. We note that the presence of the nanobodies could have reduced additional helical movements. In the MurJ structure, the two extra α-helices are proposed to function as a hydrophobic groove that binds the lipid tail of the substrate; these are absent in WzxE meaning any such groove in MurJ is not a conserved feature (figure 4*c*). In WzxE, the two small membrane helices (TM3, TM9) which are oriented parallel to the membrane (figures 1*c*, 2b and 3a) provide a hydrophobic groove which could bind the undecaprenol tail, similarly to the EH helices in PglK [85–87]. Binding to this groove would serve to fix the bulky substrate in place before, after and during translocation thus minimizing any entropic penalty associated with substrate flipping.

To determine if the volume of the lumen is directly linked to the size of the O-antigen sugar length, we compared the volume of the cavities of different WzxE and O-antigen Wzx flippase proteins, by removing any nanobodies and ligands, using the CASTp 3.0 server [76] (table 3, figure 4*d*). The WzxE protein has a cavity volume of about approximately 2200–3200 Å^3^. The cavity volume of WzxE *E. coli* in the inward-facing conformation is smaller than in the outward-facing, as expected by the clash of the Nb10 and Nb7 nanobodies that does not allow a fully open inward conformation (figure 4*d*). The Wzx proteins however seem to have a bigger cavity (approx. 2200–5700 Å^3^—table 3, figure 4*d*), compared with WzxE. This difference could be to accommodate the different substrates from three to five sugar O-antigen molecules, including branched sugars, as the different Wzx proteins from different strains and serotypes have preferred substrate specificity such as lipid III for WzxE.

In the WzxE structures, we identified two conserved arginine residues pairs, one at the periplasmic end of the lumen and the other at the cytoplasmic end (figure 2*c* and 4*a*). As a result, the central lumen is profoundly positively charged. These pairs could plausibly interact with the pyrophosphate groups of the lipid III substrate, similar to the arginine residues in the central cavity of the PglK flippase interacting with the pyrophosphate moiety of the GlcGalNAc_5_Bac-PP-undecaprenyl [86,87]. We noted that the separation of the arginine residues in each pair followed an inverse relationship with the other pair, when was one pair had a separation of 12 Å, the other pair were separated by 19 Å. R44 of the R44–R239 pair, at the periplasmic end, was shown to be essential for ECA production *in vivo* (figure 4*a*,*b*). However, we cannot exclude the possibility that the lack of ECA production could have arisen from inefficient membrane integration. The periplasmic R44–R239 pair are close to the conserved D262 (figure 4*a*,*c*). This arrangement of R44–R239–D262 is similar to the triad R24–D25–R255 in MurJ (figure 4*c*) which interacts with the pyrophosphate [39]. The carboxylate side chain would be available to coordinate any metal ion bound to the pyrophosphate of lipid III or to the sugar hydroxyls. The alignment of WzxE-Nb10-Nb7_in_ with the AlphaFold structure of Wzx_O157_ (RMSD 3.75 Å over 384 atoms, AF-A0A826NNY9-F1) shows patches of positive charged clusters at the entrance to the lumen, especially on both the cytoplasmic (arginine rich C-terminus) and periplasmic (R67, R372) surfaces of Wzx_O157_. Furthermore, the side chain of R298 from Wzx_O157_ overlaps with R44 and the residue K419 points at the same direction compared to R239 of WzxE. Those residues are essential for the Wzx_O157_ protein function [91].

### 4.1. Flipping mechanism

Noting that the C-terminal arginine cluster is vital for activity (figure 4*a*,*b*; electronic supplementary material, S1b), we propose mechanistically that the pyrophosphate group is first recognized and recruited here. The bound pyrophosphate could then migrate to the cytoplasmic arginine pair (R143, R282) in the inward-facing conformation (open to cytoplasm). We suggest that the C-terminal cluster may be sufficient for some activity, thus explaining the retention of activity for the R143A–R282A double mutant. We note a network of carboxylate residues (D71, D288, E285) that are suitably positioned to interact with the three sugar residues of ECA. Once inside the cavity, the transition of the pyrophosphate such that it now binds to the periplasmic arginine residues pair (R44, R239) is accompanied by the conformational switch to the outward-facing (open at periplasm) conformation. The conserved D262 is positioned to assist in the recognition of the sugar molecule in this location. As noted, there are several other polar residues (N42, D249, Q255) that may have a role in binding sugars. We suggest that it is the network of carboxylates and polar residues at both faces that drives sugar specificity. Once flipped to the periplasmic face, the pyrophosphate can then migrate to the positively charged arginine cluster (R315, R331) at the site marked by the sulfate molecule 433 (electronic supplementary material, figure S1b,c), before being utilized by the polymerase, WzyE. Notably, WzyE has been proposed to form a complex with WzzE [17]. By enclosing both the charged pyrophosphate and the polar sugar molecules thus shielding them from the hydrophobic lipid bilayer, WzxE reduces the activation energy required for translocation.

At a first approximation, the difference in free energy of lipid III between the inner and outer leaflet of the membrane is likely to be small, but the activation energy to translocation is very large (spontaneous translocation of lipid III does not occur). To overcome the activation energy, MOP transporters commonly utilize either ATP (PglK [87], MsbA [88]) or a Na^+^/H^+^ antiport mechanism (NorM, MurJ, DinF subfamily [22,41]). There are no nucleotide binding domains in MATE structures and no evidence that ATP directly influences activity. WzxE has been proposed to be a proton antiporter [84]. However, the positively charged central lumen seems unlikely to promote proton transport and there is no negatively charged patch that spans the membrane. Thus, we find no evidence supporting the proton antiporter hypothesis. The inward structure of MurJ_Ta_ has a chloride ion that was proposed to be essential counter-ion for the lipid II flipping mechanism [25]. However, MurJ_Ec_ has no equivalent chloride ion, indicating the counter-ion might differ between organisms [25]. Although a chloride ion was identified in one of the WzxE structures, it was located on the outside surface of the protein, a location different to that in MurJ_Ta_. Our data therefore do not support the chloride ion antiport hypothesis as a conserved mechanism for transport. The voltage difference across the membrane as possible source of activation energy has been proposed from the study of MurJ [40] but our structure does not contribute any insight to this argument.

The structural studies here are consistent with the model in which the switch between the inward- and outward-facing structures underpins the transport of the lipid-linked sugar across the inner membrane. The two conformations of WzxE revealed in this study, suggest a mechanistic role for two pairs of arginine residues, one of these pairs was shown to be required for function. We also identified an additional cluster of arginine residues that is essential. Further studies will require a substrate complex, something which we have been unable to obtain with our system. A sensitive *in vitro* assay would also aid in dissecting the contributions of individual residues to function.

## Data Availability

The crystallography structures were deposited to the Protein Data Bank (wwPDB OneDep) with the following entries 9G95, 9G97, 9G9M, 9G9N, 9G9O and 9G9P. The WzxE wild-type plasmids are deposited with ADDGENE (ADDGENE ID: 226781 and 226782). The nanobody plasmids are available at the VUB, Brussels, upon request. Supplementary material is available online [92].

## References

[1] Liston SD, Willis LM. 2021 Racing to build a wall: glycoconjugate assembly in gram-positive and gram-negative bacteria. Curr. Opin. Struct. Biol. **68**, 55–65. (10.1016/j.sbi.2020.11.013) 33429200

[2] Tytgat HLP, Lebeer S. 2014 The sweet tooth of bacteria: common themes in bacterial glycoconjugates. Microbiol. Mol. Biol. Rev. **78**, 372–417. (10.1128/MMBR.00007-14) 25184559 PMC4187687

[3] Eade CR, Wallen TW, Gates CE, Oliverio CL, Scarbrough BA, Reid AJ, Jorgenson MA, Young KD, Troutman JM. 2021 Making the enterobacterial common antigen glycan and measuring its substrate sequestration. ACS Chem. Biol. **16**, 691–700. (10.1021/acschembio.0c00983) 33740380 PMC8080848

[4] Rai AK, Mitchell AM. 2020 Enterobacterial common antigen: synthesis and function of an enigmatic molecule. MBio **11**, e01914-20. (10.1128/mBio.01914-20) 32788387 PMC7439462

[5] Lukose V, Walvoort MTC, Imperiali B. 2017 Bacterial phosphoglycosyl transferases: initiators of glycan biosynthesis at the membrane interface. Glycobiology **27**, 820–833. (10.1093/glycob/cwx064) 28810664 PMC5881778

[6] O’Toole KH, Bernstein HM, Allen KN, Imperiali B. 2021 The surprising structural and mechanistic dichotomy of membrane-associated phosphoglycosyl transferases. Biochem. Soc. Trans. **49**, 1189–1203. (10.1042/BST20200762) 34100892 PMC9206117

[7] Lehrer J, Vigeant KA, Tatar LD, Valvano MA. 2007 Functional characterization and membrane topology of Escherichia coli weca, a sugar-phosphate transferase initiating the biosynthesis of enterobacterial common antigen and O-antigen lipopolysaccharide. J. Bacteriol. **189**, 2618–2628. (10.1128/JB.01905-06) 17237164 PMC1855806

[8] Das D, Walvoort MTC, Lukose V, Imperiali B. 2016 A rapid and efficient luminescence-based method for assaying phosphoglycosyltransferase enzymes. Sci. Rep. **6**, 33412. (10.1038/srep33412) 27624811 PMC5022061

[9] Erbel PJA, Barr K, Gao N, Gerwig GJ, Rick PD, Gardner KH. 2003 Identification and biosynthesis of cyclic enterobacterial common antigen in Escherichia coli*.* J. Bacteriol. **185**, 1995–2004. (10.1128/JB.185.6.1995-2004.2003) 12618464 PMC150143

[10] Maczuga N, Tran ENH, Morona R. 2022 Subcellular localization of the enterobacterial common antigen GT-E-like glycosyltransferase, WecG. Mol. Microbiol. **118**, 403–416. (10.1111/mmi.14973) 36006410 PMC9804384

[11] Barr K, Rick PD. 1987 Biosynthesis of enterobacterial common antigen in Escherichia coli: in vitro synthesis of lipid-linked intermediates. J. Biol. Chem. **262**, 7142–7150. 3034883

[12] Barr K, Nunes-Edwards P, Rick PD. 1989 In vitro synthesis of a lipid-linked trisaccharide involved in synthesis of enterobacterial common antigen. J. Bacteriol. **171**, 1326–1332. (10.1128/jb.171.3.1326-1332.1989) 2493443 PMC209749

[13] Rick PD, Mayer H, Neumeyer BA, Wolski S, Bitter-Suermann D. 1985 Biosynthesis of enterobacterial common antigen. J. Bacteriol. **162**, 494–503. (10.1128/jb.162.2.494-503.1985) 3886625 PMC218875

[14] Liu D, Cole RA, Reeves PR. 1996 An o-antigen processing function for Wzx (RfbX): a promising candidate for o-unit flippase. J. Bacteriol. **178**, 2102–2107. (10.1128/jb.178.7.2102-2107.1996) 8606190 PMC177911

[15] Rick PD, Barr K, Sankaran K, Kajimura J, Rush JS, Waechter CJ. 2003 Evidence that the wzxE gene of Escherichia coli K-12 encodes a protein involved in the transbilayer movement of a trisaccharide-lipid intermediate in the assembly of enterobacterial common antigen. J. Biol. Chem. **278**, 16534–16542. (10.1074/jbc.M301750200) 12621029

[16] Maczuga NT, Tran ENH, Morona R. 2022 Topology of the Shigella flexneri enterobacterial common antigen polymerase WzyE. Microbiology (NY) **168**. (10.1099/mic.0.001183) 35470793

[17] Weckener M, Woodward LS, Clarke BR, Liu H, Ward PN, Le Bas A, Bhella D, Whitfield C, Naismith JH. 2023 The lipid linked oligosaccharide polymerase Wzy and its regulating co-polymerase, Wzz, from enterobacterial common antigen biosynthesis form a complex. Open Biol. **13**. (10.1098/rsob.220373) PMC10030265 36944376

[18] Dell A, Oates J, Lugowski C, Romanowska E, Kenne L, Lindberg B. 1984 The enterobacterial common-antigen, a cyclic polysaccharide. Carbohydr. Res. **133**, 95–104. (10.1016/0008-6215(84)85186-1) 6498860

[19] Morris KN, Mitchell AM. 2023 Phosphatidylglycerol is the lipid donor for synthesis of phospholipid-linked enterobacterial common antigen. J. Bacteriol. **205**, e0040322. (10.1128/jb.00403-22) 36622229 PMC9879101

[20] Ashraf KU *et al*. 2022 Structural basis of lipopolysaccharide maturation by the O-antigen ligase. Nature **604**, 371–376. (10.1038/s41586-022-04555-x) 35388216 PMC9884178

[21] Ruiz N, Kahne D, Silhavy TJ. 2009 Transport of lipopolysaccharide across the cell envelope: the long road of discovery. Nat. Rev. Microbiol. **7**, 677–683. (10.1038/nrmicro2184) 19633680 PMC2790178

[22] Hvorup RN, Winnen B, Chang AB, Jiang Y, Zhou XF, Saier MH. 2003 The multidrug/oligosaccharidyl-lipid/polysaccharide (MOP) exporter superfamily. Eur. J. Biochem. **270**, 799–813. (10.1046/j.1432-1033.2003.03418.x) 12603313

[23] Hong Y, Liu MA, Reeves PR. 2018 Progress in our understanding of Wzx flippase for translocation of bacterial membrane lipid-linked oligosaccharide. J. Bacteriol. **200**, e00154-17. (10.1128/JB.00154-17) 28696276 PMC5717161

[24] Kuk ACY, Hao A, Lee SY. 2022 Structure and mechanism of the lipid flippase MurJ. Annu. Rev. Biochem. **91**, 705–729. (10.1146/annurev-biochem-040320-105145) 35320686 PMC10108830

[25] Kumar S, Mollo A, Rubino FA, Kahne D, Ruiz N. 2023 Chloride ions are required for Thermosipho africanus MurJ function. MBio **14**, e0008923. (10.1128/mbio.00089-23) 36752629 PMC9973255

[26] Marolda CL, Vicarioli J, Valvano MA. 2004 Wzx proteins involved in biosynthesis of O antigen function in association with the first sugar of the O-specific lipopolysaccharide subunit. Microbiology (NY) **150**, 4095–4105. (10.1099/mic.0.27456-0) 15583162

[27] Feldman MF, Marolda CL, Monteiro MA, Perry MB, Parodi AJ, Valvano MA. 1999 The activity of a putative polyisoprenol-linked sugar translocase (Wzx) involved in Escherichia coli O antigen assembly is independent of the chemical structure of the O repeat. J. Biol. Chem. **274**, 35129–35138. (10.1074/jbc.274.49.35129) 10574995

[28] Marolda CL, Tatar LD, Alaimo C, Aebi M, Valvano MA. 2006 Interplay of the Wzx translocase and the corresponding polymerase and chain length regulator proteins in the translocation and periplasmic assembly of lipopolysaccharide O antigen. J. Bacteriol. **188**, 5124–5135. (10.1128/JB.00461-06) 16816184 PMC1539953

[29] Liu MA, Morris P, Reeves PR. 2019 Wzx flippases exhibiting complex O-unit preferences require a new model for Wzx-substrate interactions. Microbiologyopen **8**, e00655. (10.1002/mbo3.655) 29888516 PMC6436433

[30] Hong Y, Reeves PR. 2014 Diversity of O-antigen repeat unit structures can account for the substantial sequence variation of Wzx translocases. J. Bacteriol. **196**, 1713–1722. (10.1128/JB.01323-13) 24532778 PMC3993327

[31] Hong Y, Hu D, Verderosa AD, Qin J, Totsika M, Reeves PR. 2023 Repeat-unit elongations to produce bacterial complex long polysaccharide chains, an O-antigen perspective. EcoSal Plus **11**, eesp00202022. (10.1128/ecosalplus.esp-0020-2022) 36622162 PMC10729934

[32] Islam ST, Fieldhouse RJ, Anderson EM, Taylor VL, Keates RAB, Ford RC, Lam JS. 2012 A cationic lumen in the Wzx flippase mediates anionic O-antigen subunit translocation in Pseudomonas aeruginosa PAO1. Mol. Microbiol. **84**, 1165–1176. (10.1111/j.1365-2958.2012.08084.x) 22554073 PMC3412221

[33] Lu M. 2016 Structures of multidrug and toxic compound extrusion transporters and their mechanistic implications. Channels **10**, 88–100. (10.1080/19336950.2015.1106654) 26488689 PMC4960993

[34] Lu M, Symersky J, Radchenko M, Koide A, Guo Y, Nie R, Koide S. 2013 Structures of a Na^+-^coupled, substrate-bound MATE multidrug transporter. Proc. Natl Acad. Sci. USA **110**, 2099–2104. (10.1073/pnas.1219901110) 23341609 PMC3568332

[35] He X, Szewczyk P, Karyakin A, Evin M, Hong WX, Zhang Q, Chang G. 2010 Structure of a cation-bound multidrug and toxic compound extrusion transporter. Nature **467**, 991–994. (10.1038/nature09408) 20861838 PMC3152480

[36] Bloch JS, Mukherjee S, Kowal J, Filippova EV, Niederer M, Pardon E, Steyaert J, Kossiakoff AA, Locher KP. 2021 Development of a universal nanobody-binding fab module for fiducial-assisted cryo-EM studies of membrane proteins. Proc. Natl Acad. Sci. USA **118**, e2115435118. (10.1073/pnas.2115435118) 34782475 PMC8617411

[37] Jumper J *et al*. 2021 Highly accurate protein structure prediction with alphafold. Nature **596**, 583–589. (10.1038/s41586-021-03819-2) 34265844 PMC8371605

[38] Varadi M *et al*. 2022 AlphaFold protein structure database: massively expanding the structural coverage of protein-sequence space with high-accuracy models. Nucleic Acids Res. **50**, D439–D444. (10.1093/nar/gkab1061) 34791371 PMC8728224

[39] Kuk ACY, Mashalidis EH, Lee SY. 2017 Crystal structure of the MOP flippase MurJ in an inward-facing conformation. Nat. Struct. Mol. Biol. **24**, 171–176. (10.1038/nsmb.3346) 28024149 PMC5382020

[40] Zheng S, Sham LT, Rubino FA, Brock KP, Robins WP, Mekalanos JJ, Marks DS, Bernhardt TG, Kruse AC. 2018 Structure and mutagenic analysis of the lipid II flippase MurJ from Escherichia coli. Proc. Natl Acad. Sci. USA **115**, 6709–6714. (10.1073/pnas.1802192115) 29891673 PMC6042122

[41] Kusakizako T, Miyauchi H, Ishitani R, Nureki O. 2020 Structural biology of the multidrug and toxic compound extrusion superfamily transporters. Biochim. et Biophys. Acta (BBA)—Biomembrane. **1862**, 183154. (10.1016/j.bbamem.2019.183154) 31866287

[42] Kohga H, Mori T, Tanaka Y, Yoshikaie K, Taniguchi K, Fujimoto K, Fritz L, Schneider T, Tsukazaki T. 2022 Crystal structure of the lipid flippase MurJ in a ‘squeezed’ form distinct from its inward- and outward-facing forms. Structure **30**, 1088–1097.(10.1016/j.str.2022.05.008) 35660157

[43] Kuk ACY, Hao A, Guan Z, Lee SY. 2019 Visualizing conformation transitions of the lipid II flippase MurJ. Nat. Commun. **10**, 1736. (10.1038/s41467-019-09658-0) 30988294 PMC6465408

[44] Sham LT, Zheng S, Yakhnina AA, Kruse AC, Bernhardt TG. 2018 Loss of specificity variants of WzxC suggest that substrate recognition is coupled with transporter opening in MOP-family flippases. Mol. Microbiol. **109**, 633–641. (10.1111/mmi.14002) 29907971 PMC6181778

[45] Liu H, Naismith JH. 2008 An efficient one-step site-directed deletion, insertion, single and multiple-site plasmid mutagenesis protocol. BMC Biotechnol. **8**, 91. (10.1186/1472-6750-8-91) 19055817 PMC2629768

[46] Neuhard J, Thomassen E. 1976 Altered deoxyribonucleotide pools in P2 eductants of Escherichia coli K-12 due to deletion of the dcd gene. J. Bacteriol. **126**, 999–1001. (10.1128/jb.126.2.999-1001.1976) 177407 PMC233240

[47] Sawitzke JA, Thomason LC, Costantino N, Bubunenko M, Datta S, Court DL. 2007 Recombineering: in vivo genetic engineering in E. coli, S. enterica, and beyond. Meth. Enzymol. **421**, 171–199. (10.1016/S0076-6879(06)21015-2) 17352923

[48] Guzman LM, Belin D, Carson MJ, Beckwith J. 1995 Tight regulation, modulation, and high-level expression by vectors containing the arabinose PBAD promoter. J. Bacteriol. **177**, 4121–4130. (10.1128/jb.177.14.4121-4130.1995) 7608087 PMC177145

[49] Miroux B, Walker JE. 1996 Over-production of proteins in Escherichia coli: mutant hosts that allow synthesis of some membrane proteins and globular proteins at high levels. J. Mol. Biol. **260**, 289–298. (10.1006/jmbi.1996.0399) 8757792

[50] Pardon E *et al*. 2014 A general protocol for the generation of nanobodies for structural biology. Nat. Protoc. **9**, 674–693. (10.1038/nprot.2014.039) 24577359 PMC4297639

[51] El Omari K *et al*. 2023 Experimental phasing opportunities for macromolecular crystallography at very long wavelengths. Commun. Chem. **6**, 219. (10.1038/s42004-023-01014-0) 37828292 PMC10570326

[52] Kabsch W. 2010 XDS. Acta. Cryst. D. Biol. Cryst. **66**, 125–132. (10.1107/S0907444909047337) PMC2815665 20124692

[53] Skubák P, Pannu NS. 2013 Automatic protein structure solution from weak X-ray data. Nat. Commun. **4**, 2777. (10.1038/ncomms3777) 24231803 PMC3868232

[54] Winn MD *et al*. 2011 Overview of the CCP4 suite and current developments. Acta. Cryst. D. Biol. Cryst. **67**, 235–242. (10.1107/S0907444910045749) PMC3069738 21460441

[55] Terwilliger TC, Adams PD, Read RJ, McCoy AJ, Moriarty NW, Grosse-Kunstleve RW, Afonine PV, Zwart PH, Hung LW. 2009 Decision-making in structure solution using Bayesian estimates of map quality: the PHENIX AutoSol wizard. Acta Crystallogr. D Biol. Crystallogr. **65**, 582–601. (10.1107/S0907444909012098) 19465773 PMC2685735

[56] Liebschner D *et al*. 2019 Macromolecular structure determination using X-rays, neutrons and electrons: recent developments in phenix. Acta Crystallogr. D. Struct. Biol. **75**, 861–877. (10.1107/S2059798319011471) 31588918 PMC6778852

[57] Cowtan K. 2006 The buccaneer software for automated model building. 1. Tracing protein chains. Acta. Cryst. D. Biol. Cryst. **62**, 1002–1011. (10.1107/S0907444906022116) 16929101

[58] Winter G. 2010 Xia2: an expert system for macromolecular crystallography data reduction. J. Appl. Cryst. **43**, 186–190. (10.1107/S0021889809045701)

[59] McCoy AJ, Grosse-Kunstleve RW, Adams PD, Winn MD, Storoni LC, Read RJ. 2007 Phaser crystallographic software. J. Appl. Crystallogr. **40**, 658–674. (10.1107/S0021889807021206) 19461840 PMC2483472

[60] Murshudov GN, Skubák P, Lebedev AA, Pannu NS, Steiner RA, Nicholls RA, Winn MD, Long F, Vagin AA. 2011 REFMAC5 for the refinement of macromolecular crystal structures. Acta. Cryst. D. Biol. Cryst. **67**, 355–367. (10.1107/S0907444911001314) PMC3069751 21460454

[61] Williams CJ *et al*. 2018 MolProbity: more and better reference data for improved all-atom structure validation. Protein Sci. **27**, 293–315. (10.1002/pro.3330) 29067766 PMC5734394

[62] Pettersen EF, Goddard TD, Huang CC, Couch GS, Greenblatt DM, Meng EC, Ferrin TE. 2004 UCSF Chimera—a visualization system for exploratory research and analysis. J. Comput. Chem. **25**, 1605–1612. (10.1002/jcc.20084) 15264254

[63] Laemmli UK. 1970 Cleavage of structural proteins during the assembly of the head of bacteriophage T4. Nature **227**, 680–685. (10.1038/227680a0) 5432063

[64] Bateman A *et al*. 2023 UniProt: the universal protein knowledgebase in 2023. Nucleic Acids Res. **51**, D523–D531. (10.1093/nar/gkac1052) 36408920 PMC9825514

[65] Madeira F, Madhusoodanan N, Lee J, Eusebi A, Niewielska A, Tivey ARN, Lopez R, Butcher S. 2024 The EMBL-EBI job dispatcher sequence analysis tools framework in 2024. Nucleic Acids Res. **52**, W521–W525. (10.1093/nar/gkae241) 38597606 PMC11223882

[66] Waterhouse AM, Procter JB, Martin DMA, Clamp M, Barton GJ. 2009 Jalview version 2—a multiple sequence alignment editor and analysis workbench. Bioinformatics **25**, 1189–1191. (10.1093/bioinformatics/btp033) 19151095 PMC2672624

[67] Robert X, Gouet P. 2014 Deciphering key features in protein structures with the new endscript server. Nucleic Acids Res. **42**, W320–4. (10.1093/nar/gku316) 24753421 PMC4086106

[68] Islam ST, Taylor VL, Qi M, Lam JS. 2010 Membrane topology mapping of the o-antigen flippase (Wzx), polymerase (Wzy), and ligase (Waal) from Pseudomonas aeruginosa PAO1 reveals novel domain architectures. MBio **1**, e00189-10. (10.1128/mBio.00189-10) 20824106 PMC2932511

[69] Khemaissa S, Sagan S, Walrant A. Tryptophan, an amino-acid endowed with unique properties and its many roles in membrane proteins. Crystals (Basel) **11**, 1032. (10.3390/cryst11091032)

[70] Liu B, Knirel YA, Feng L, Perepelov AV, Senchenkova SN, Wang Q, Reeves PR, Wang L. 2008 Structure and genetics of Shigella O antigens. FEMS Microbiol. Rev. **32**, 627–653. (10.1111/j.1574-6976.2008.00114.x) 18422615

[71] Stevenson G, Andrianopoulos K, Hobbs M, Reeves PR. 1996 Organization of the Escherichia coli K-12 gene cluster responsible for production of the extracellular polysaccharide colanic acid. J. Bacteriol. **178**, 4885–4893. (10.1128/jb.178.16.4885-4893.1996) 8759852 PMC178271

[72] Rocchetta HL, Burrows LL, Lam JS. 1999 Genetics of O-antigen biosynthesis in Pseudomonas aeruginosa. Microbiol. Mol. Biol. Rev. **63**, 523–553. (10.1128/MMBR.63.3.523-553.1999) 10477307 PMC103745

[73] Islam ST, Lam JS. 2014 Synthesis of bacterial polysaccharides via the Wzx/Wzy-dependent pathway. Can. J. Microbiol. **60**, 697–716. (10.1139/cjm-2014-0595) 25358682

[74] Hong Y, Cunneen MM, Reeves PR. 2012 The Wzx translocases for Salmonella enterica o-antigen processing have unexpected serotype specificity. Mol. Microbiol. **84**, 620–630. (10.1111/j.1365-2958.2012.08048.x) 22497246

[75] Drummelsmith J, Whitfield C. 1999 Gene products required for surface expression of the capsular form of the group 1 k antigen in Escherichia coli (O9a:K30). Mol. Microbiol. **31**, 1321–1332. (10.1046/j.1365-2958.1999.01277.x) 10200954

[76] Tian W, Chen C, Lei X, Zhao J, Liang J. 2018 CASTp 3.0: computed atlas of surface topography of proteins. Nucleic Acids Res. **46**, W363–W367. (10.1093/nar/gky473) 29860391 PMC6031066

[77] Jorgenson MA, Young KD. 2016 Interrupting biosynthesis of O antigen or the lipopolysaccharide core produces morphological defects in escherichia coli by sequestering undecaprenyl phosphate. J. Bacteriol. **198**, 3070–3079. (10.1128/JB.00550-16) 27573014 PMC5075036

[78] Jorgenson MA, Kannan S, Laubacher ME, Young KD. 2016 Dead-end intermediates in the enterobacterial common antigen pathway induce morphological defects in Escherichia coli by competing for undecaprenyl phosphate. Mol. Microbiol. **100**, 1–14. (10.1111/mmi.13284) 26593043 PMC4845916

[79] Rush JS, Alaimo C, Robbiani R, Wacker M, Waechter CJ. 2010 A novel epimerase that converts GlcNAc-P-P-undecaprenol to GalNAc-P-P-undecaprenol in Escherichia coli 0157. J. Biol. Chem. **285**, 1671–1680. (10.1074/jbc.M109.061630) 19923219 PMC2804325

[80] Miyauchi H *et al*. 2017 Structural basis for xenobiotic extrusion by eukaryotic mate transporter. Nat. Commun. **8**, 1633. (10.1038/s41467-017-01541-0) 29158478 PMC5696359

[81] Raturi S, Nair AV, Shinoda K, Singh H, Bai B, Murakami S, Fujitani H, van Veen HW. 2021 Engineered MATE multidrug transporters reveal two functionally distinct ion-coupling pathways in NorM from Vibrio Cholerae. Commun. Biol. **4**, 558. (10.1038/s42003-021-02081-6) 33976372 PMC8113278

[82] Zakrzewska S *et al*. 2019 Inward-facing conformation of a multidrug resistance mate family transporter. Proc. Natl Acad. Sci. USA **116**, 12275–12284. (10.1073/pnas.1904210116) 31160466 PMC6589766

[83] Islam ST, Lam JS. 2013 Wzx flippase-mediated membrane translocation of sugar polymer precursors in bacteria. Environ. Microbiol. **15**, 1001–1015. (10.1111/j.1462-2920.2012.02890.x) 23016929

[84] Islam ST, Eckford PDW, Jones ML, Nugent T, Bear CE, Vogel C, Lam JS. 2013 Proton-dependent gating and proton uptake by Wzx support O-antigen-subunit antiport across the bacterial inner membrane. MBio **4**, e00678-13. (10.1128/mBio.00678-13) 24023388 PMC3774195

[85] Perez C, Mehdipour AR, Hummer G, Locher KP. 2019 Structure of outward-facing pglk and molecular dynamics of lipid-linked oligosaccharide recognition and translocation. Structure **27**, 669–678.(10.1016/j.str.2019.01.013) 30799077

[86] Perez C, Köhler M, Janser D, Pardon E, Steyaert J, Zenobi R, Locher KP. 2017 Structural basis of inhibition of lipid-linked oligosaccharide flippase PglK by a conformational nanobody. Sci. Rep. **7**, 46641. (10.1038/srep46641) 28422165 PMC5395944

[87] Perez C, Gerber S, Boilevin J, Bucher M, Darbre T, Aebi M, Reymond JL, Locher KP. 2015 Structure and mechanism of an active lipid-linked oligosaccharide flippase. Nature **524**, 433–438. (10.1038/nature14953) 26266984

[88] Mi W, Li Y, Yoon SH, Ernst RK, Walz T, Liao M. 2017 Structural basis of MsbA-mediated lipopolysaccharide transport. Nature **549**, 233–237. (10.1038/nature23649) 28869968 PMC5759761

[89] Padayatti PS, Lee SC, Stanfield RL, Wen PC, Tajkhorshid E, Wilson IA, Zhang Q. 2019 Structural insights into the lipid a transport pathway in MsbA. Structure **27**, 1114–1123.(10.1016/j.str.2019.04.007) 31130486 PMC6610721

[90] Bi Y, Mann E, Whitfield C, Zimmer J. 2018 Architecture of a channel-forming O-antigen polysaccharide ABC transporter. Nature **553**, 361–365. (10.1038/nature25190) 29320481 PMC5978415

[91] Marolda CL, Li B, Lung M, Yang M, Hanuszkiewicz A, Rosales AR, Valvano MA. 2010 Membrane topology and identification of critical amino acid residues in the Wzx O-antigen translocase from Escherichia coli 0157:H4. J. Bacteriol. **192**, 6160–6171. (10.1128/JB.00141-10) 20870764 PMC2981206

[92] Audrey LB, Clarke BR, Teelucksingh T, Lee M, Kamel EO, Giltrap AM *et al*. 2024 Supplementary material from: Structure of WzxE the lipid III flippase for Enterobacterial Common Antigen polysaccharide. Figshare. (10.6084/m9.figshare.c.7587811) PMC11706664 39772807

